# Multi‐Omics Profiling of the *Scaphoideus titanus* Yeast‐Like Symbiont Guides the Bioinformatic Discovery of Related Fungal Symbioses in Insects

**DOI:** 10.1111/1462-2920.70361

**Published:** 2026-07-02

**Authors:** Simona Abbà, Marta Vallino, Alessandro Cicerone, Simona Cirrincione, Beatrice Aiuto, Luciana Galetto, Marika Rossi

**Affiliations:** ^1^ Istituto per la Protezione Sostenibile delle Piante Consiglio Nazionale delle Ricerche, IPSP‐CNR Turin Italy; ^2^ Dipartimento di Scienze Agrarie, Forestali ed Alimentari Università degli Studi di Torino Grugliasco Torino Italy; ^3^ Istituto di Scienze delle Produzioni Alimentari Consiglio Nazionale delle Ricerche, ISPA‐CNR Grugliasco Italy

**Keywords:** data mining, hemiptera, microbiome, *Ophiocordyceps*, YLS

## Abstract

Symbiotic partnerships have opened new ecological niches and contributed to the remarkable diversification of insects. The leafhopper *Scaphoideus titanus*, a phloem‐feeding insect known to be the primary vector of Flavescence dorée phytoplasma, harbours two primary endosymbionts: the bacterium ‘*Candidatus* Karelsulcia muelleri’ and a yeast‐like symbiont (YLS). While most studies on insect‐associated microorganisms have focused on obligate bacterial symbionts, fungal endosymbionts, although documented for almost a century, are only now gaining renewed attention for their evolutionary and ecological significance. In this study, we integrated genomic and proteomic data with phylogenetic analyses to elucidate the functional and evolutionary features of the YLS associated with *S. titanus*. Using a data‐independent proteomic approach supported by a newly sequenced symbiont genome, we defined the proteins expressed by the YLS that may contribute to host physiology. Comparative analyses across the five currently available YLS genomes enabled a proteome‐wide phylogenetic reconstruction within the genus *Ophiocordyceps*, refining the evolutionary placement of these symbioses. Finally, large‐scale mining of NCBI transcriptomic Sequence Read Archive datasets using a novel computational workflow, combined with an extensive literature survey, identified several new candidate insect hosts and provided a comprehensive inventory of species harbouring these fungal partners.

## Graphical abstract

Genomic, proteomic and phylogenetic analyses of the obligate yeast‐like symbiont (YLS) associated with *Scaphoideus titanus* revealed retained metabolic flexibility and a monophyletic origin of insect‐associated YLSs within a free‐living fungal lineage. A novel computational pipeline was used to mine NCBI transcriptomic data and identified new candidate insect hosts for these fungi.

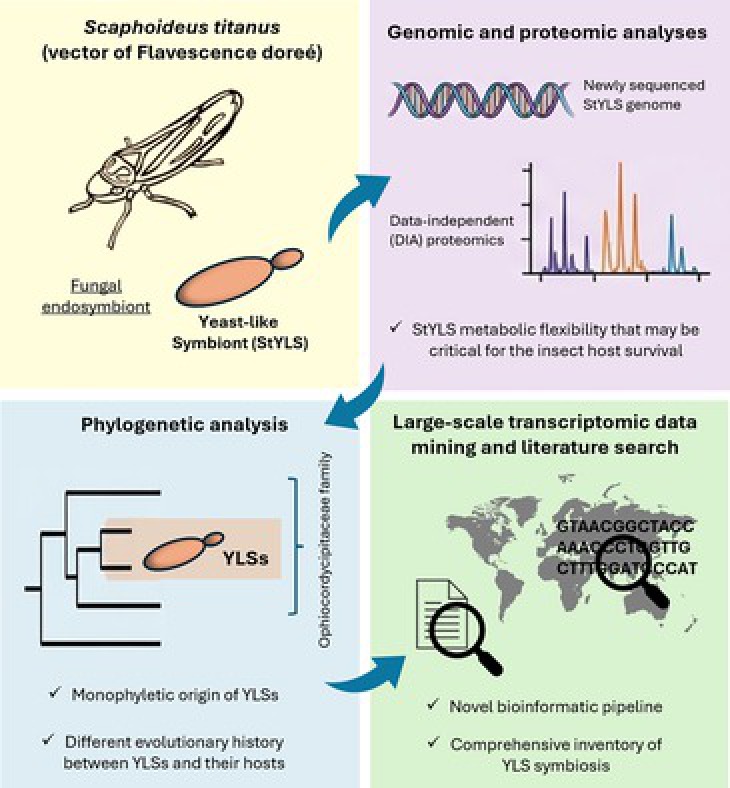

## 1 Introduction

Yeast symbionts are fundamental to insect ecology and evolution, influencing nutrition on recalcitrant food (Ali et al. 2017; Toki 2021), development (Valzania et al. 2018; Bellutti et al. 2018), immunity (Meriggi et al. 2019; Malassigné et al. 2021) and the ability to exploit nutrient‐poor niches (Malassigné et al. 2021; Noda and Koizumi 2003). These fungal partners include both true yeasts (Ascomycota: Saccharomycotina) and yeast‐like symbionts (YLSs) (Ascomycota: Pezizomycotina), which represent lineages of filamentous fungi that evolved a yeast‐like budding morphology in association with their insect hosts (Blackwell 2017). Yeasts exhibit a broad spectrum of dependency on their insect hosts, ranging from facultative associations to obligate mutualism. Despite the taxonomic diversity of both insects and yeasts, these fungi often perform similar functional roles across different insect orders, indicating functional convergence in their associations. In facultative opportunistic associations, yeasts usually provide volatile compounds that attract insects to substrates, whereas insects function as vectors carrying yeasts from one substrate to another. Such interactions are well documented in nitidulid beetles (Coleoptera: Nitidulidae) (Baig et al. 2023), in armyworm moths (Lepidoptera: Noctuidae) (Ma et al. 2025) and in species of *Drosophila* (Diptera: Drosophilidae) (Becher et al. 2012), where yeast‐derived attractants strongly influence host foraging and oviposition behaviour.

At a higher level of integration, some mutualisms are characterized by vertical transmission and the evolution of specialized organs (e.g., mycetomes) that house yeast symbionts essential for insect development and survival. In the stable mutual dependence between species of the yeast‐like fungus *Symbiotaphrina* and anobiid beetles (Coleoptera: Anobiidae), the fungus detoxifies recalcitrant compounds in the insect diet and provides essential nutrients, including B‐group vitamins and sterols (Martinson 2020). In return, the fungal symbiont gains access to a stable, nutrient‐rich environment that protects it from external stressors and competition from other microbes.

The most extreme forms of specialization occur where YLSs lose the ability to exist independently and become fully interdependent with their insect hosts, a phenomenon exemplified by the YLS associated with many species of cicadas, soft scale insects, planthoppers and leafhoppers (Zhou et al. 2025; Gomez‐Polo et al. 2017; Matsuura et al. 2018; Szklarzewicz et al. 2021; Vogel and Moran 2013; Ward et al. 2024; Nishino et al. 2016; Vaishally et al. 2025; Sasaki et al. 1996; Sacchi et al. 2008). Almost all these fungal symbionts have become so profoundly integrated into host biology that they function as primary symbionts and can no longer complete their life cycles outside the insect. Such hemipterans, in turn, are dependent on them particularly for the provision of essential amino acids, B‐group vitamins and sterols, which are typically deficient in their diet. YLSs are most commonly localized in the fat bodies and haemolymph of their insect hosts (Matsuura et al. 2018; Kobiałka, Michalik, Walczak, and Szklarzewicz 2018; Szklarzewicz and Michalik 2017; Vashishtha et al. 2011; Podsiadło et al. 2018). However, in some cicadas, they also colonize the bacteriomes, where they may coexist with bacterial endosymbionts (Matsuura et al. 2018; Huang et al. 2024; Hill et al. 2021; Wang et al. 2021).

Their phylogenetic relationship with fungi belonging to the order Hypocreales has been demonstrated since the earliest molecular reports (Noda et al. 1995). However, the identity of the most closely related species has varied across studies of different insect hosts. Recent phylogenetic revisions and omics‐based studies have reconciled many of these discrepancies and placed fungal endosymbionts of cicadas and soft scales in the same clade as the genus *Ophiocordyceps*. The most plausible hypothesis for such phylogenetic relationships is that these fungal endosymbionts may originate from entomopathogenic fungi that lost their virulence and became domesticated and vertically transmitted (Suh et al. 2001).

In this study, we integrated genomic and proteomic data with a comprehensive phylogenetic analysis to reconstruct the functional and evolutionary framework of the YLS associated with a leafhopper of agronomical interest, *Scaphoideus titanus* (Hemiptera: Cicadellidae), the primary vector of Flavescence dorée phytoplasma in grapevines, a severe threat to viticulture in Europe. This insect harbours a YLS, hereafter referred to as StYLS, and the bacterium ‘*Candidatus* Karelsulcia muelleri’ (Bacteroidota, hereafter *Karelsulcia*) as its primary symbionts (Sacchi et al. 2008; Abbà et al. 2022). In addition, it is associated with a variable population of facultative symbionts, the most stable and prevalent of which in European populations is ‘*Candidatus* Cardinium’ (Bacteroidota, hereafter *Cardinium*) (Sacchi et al. 2008).

Using Data‐Independent Acquisition (DIA) proteomics supported by a newly sequenced genome, we identified proteins actively expressed by the fungal endosymbiont that may have metabolic relevance for *S. titanus*. In addition, by comparing the other five YLS genomes available so far, we have performed a proteome‐wide phylogenetic analysis to better resolve the evolutionary origin of these symbioses. Finally, we placed this symbiosis in a broader context by mining NCBI transcriptomic Sequence Read Archive (SRA) datasets using a newly developed two‐step pipeline. This extensive bioinformatic survey identified novel putative YLS‐insect associations, significantly expanding the known host range beyond previous reports.

## 2 Materials and Methods

### 2.1 *Scaphoideus titanus* Collection and Plant Rearing

A *Scaphoideus titanus* colony was reared under greenhouse conditions, starting with eggs laid in grapevine canes collected in February 2024 from vineyards in Revello, Italy (44°39′20″ N, 7°23′33″ E), where *S. titanus* adults had been abundant in the previous summer. The canes bearing eggs were initially stored in a cold room at 6°C ± 1°C. When required, they were transferred to an insect‐proof greenhouse and maintained under controlled conditions (*T* = 22°C ± 3°C, photoperiod 16:8 L:D). Following a period of 3 weeks, the canes were transferred to insect‐proof mesh cages containing broad bean ( *Vicia faba* ) plants and grapevine ( *Vitis vinifera* , cultivar Barbera) grafted cuttings as food sources for emerging *S. titanus* nymphs. Broad bean and grapevine plants were replaced every 3 weeks.

### 2.2 Attempts to Cultivate StYLS

To cultivate StYLS, *S. titanus* adults were surface‐sterilized by two washes in 70% ethanol, followed by two washes in 1× phosphate‐buffered saline (PBS). The insects were dissected, and the fat bodies were isolated and homogenized in sterile 1× PBS. The homogenates were observed under a DM750 (Leica) optical microscope equipped with a Leica EC4 camera to confirm the presence of yeast‐like symbionts (YLS) and were subsequently plated onto the media used by Matsuura and colleagues for the isolation of YLS from various species of cicadas (Matsuura et al. 2018). Sabouraud dextrose agar amended with 20 μg/mL chloramphenicol was also used either alone or supplemented with yeast extract (10 g/L), egg yolk (3 yolks/500 mL) or lyophilized and grinded *Scaphoideus titanus* insects (1% w/v). Plates were incubated at 26°C for up to 8 weeks.

### 2.3 DNA Extraction and Sequencing

Genomic DNA was extracted from a pool of 30 surface‐sterilized *S. titanus* individuals. To enrich the endosymbiont population, each insect was dissected to remove the head, thorax and legs; only the abdomens, containing the digestive organs and associated fat bodies, were retained. Tissues were homogenized in 1.5 mL microcentrifuge tubes containing 1× PBS and sterile sand using a plastic pestle to facilitate disruption. The resulting homogenate was adjusted to a final volume of 500 μL with 1× PBS and filtered through a 40 μm cell strainer (Corning, USA) to remove chitinous debris.

The filtrate was centrifuged at 13,000 rpm for 3 min, and the pellet was resuspended in Sorbitol‐Citrate‐EDTA‐mercaptoethanol (SCEM) buffer (1 M sorbitol, 0.1 M sodium citrate, 10 mM EDTA, pH 8.0). To generate fungal spheroplasts, the suspension was supplemented with lyticase (Merck KGaA, Germany) to a final concentration of 400 U/mL and β‐mercaptoethanol to a final concentration of 2.1 μL/mL, followed by incubation for 2 h at 37°C. After incubation, the samples were centrifuged at 5000× *g* for 10 min at 4°C. The pellet was resuspended in 500 μL of G2 digestion buffer (Qiagen, Germany) supplemented with RNase A and Proteinase K (final concentration 450 μg/mL) and incubated at 50°C for 30 min. Genomic DNA was purified using a standard phenol–chloroform–isoamyl alcohol extraction protocol. DNA was precipitated with 2.5 M ammonium acetate and two volumes of 100% ethanol, washed with cold 70% ethanol, and air‐dried. The final pellet was resuspended in 25 μL of 10 mM Tris–HCl (pH 8.2). A total of 1.3 μg of DNA was obtained, and its quality and quantity were assessed using a Qubit fluorometer (Thermo Fisher Scientific, USA) and agarose gel electrophoresis.

The DNA was submitted to IGA Technology Services (Udine, Italy) for library preparation, and sequencing. The Celero DNA‐Seq library prep kit (Tecan, Männedorf, Switzerland) was used for library preparation and sequencing was performed on NovaSeqX (San Diego, CA, USA) in paired‐end 150 bp mode, generating approximately 3.52 billion raw reads.

### 2.4 Genome Assembly and Annotation

Illumina reads were first subjected to quality control using the BBTools suite (Bushnell B., sourceforge.net/projects/bbmap/) to remove adapter sequences, low‐quality bases and potential contaminants.

As the sequenced sample consisted of a mixture of DNA from the insect host, *Karelsulcia muelleri* and StYLS, the dataset was subjected to an initial depletion of insect and bacterial reads using *Scaphoideus titanus* transcriptome assembly TSA GJQL00000000.1 (NCBI BioProject PRJNA765507). The remaining reads were assembled using SPAdes v4.2.0 in ‘meta’ mode (Nurk et al. 2017). Draft assemblies were combined with RagTag v2.1 (Alonge et al. 2022), scaffolded using SSPACE v2 (Boetzer et al. 2011) and subjected to CDS and protein prediction using TransDecoder (https://github.com/TransDecoder/TransDecoder/) (Haas 2023). The predicted proteins were analysed using BLASTp against the NCBI core‐nr (non‐redundant) database (November 2025 release). Based on the resulting hits, the scaffolds from which the proteins were predicted were classified as insect‐, bacterial‐ or fungal‐derived.

Scaffolds of fungal origin were re‐annotated using the MAKER annotation pipeline (v. 2.31.11) (Cantarel et al. 2008) and Funannotate (v. 1.8.17) (Palmer and Stajich 2020). The annotations for both tools were based on transcripts derived from NCBI BioProject PRJNA765507 (Abbà et al. 2022). The MAKER pipeline was configured to utilize the Dfam database (bundled with MAKER) as the primary source for transposable element profiles.

MAKER was executed in a second round, integrating gene predictions from the initial MAKER and Funannotate runs using the phylogenetically closest available species, *Fusarium oxysporum*, as the model organism for ab initio gene prediction. Final gene models were extracted with gffread and used to train a species‐specific AUGUSTUS ab initio gene predictor (v. 3.4.0). This trained model was then employed in a final MAKER run to generate the definitive set of coding sequences and predicted protein products.

Functional annotation of all predicted protein‐coding sequences was performed using BLASTp analysis against the NCBI core‐nr database (threshold: Evalue < 0.0001). The predicted proteome was further analysed using BlastKOALA and KEGG Automatic Annotation Server (KAAS) for draft genomes to assign KEGG Orthology (KO) identifiers and assess the completeness of KEGG pathways.

To identify Carbohydrate‐Active Enzymes (CAzymes), the predicted proteome of StYLS was analysed using the dbCAN pipeline (v5.2.6) (Yin et al. 2012). Protein sequences were searched against the dbCAN HMM database (v12) using HMMER3, the CAZy database using DIAMOND, and the substrate‐specific dbCAN‐sub database. Following the standard dbCAN consensus criteria, a protein was conservatively annotated as a CAzyme only if it was simultaneously identified by all three tools. Additionally, signal peptides were predicted using SignalP 6.0 within the dbCAN pipeline to differentiate between intracellular enzymes and the secreted ones.

### 2.5 BUSCO Predictions and Phylogenetic Analysis

The completeness of the newly assembled StYLS genome was assessed using BUSCO v6 (lineage: hypocreales_odb12) (Manni et al. 2021). Furthermore, BUSCO v6 was also used to identify the *Hypocreales* core proteins of the other five available YLS genomes, that is, the YLSs of *Meimuna opalifera*, *Kerria lacca*, *Parthenolecanium corni*, *Nilaparvata lugens* and *Cerataphis brasiliensis* (Data S1). Due to the fragmentation of some genomes, when multiple copies of core proteins were found, only the copy with the HMMER best bit score was used in subsequent analyses. All available Ophiocordycipitaceae proteomes with scaffold‐level assemblies, along with the proteomes of other members of Hypocreales, were retrieved from NCBI and analysed using the same criteria (Data S1).

The core proteins of the selected species were used to infer phylogenetic relationships using Phylophlan v3 (Asnicar et al. 2020). Multiple sequence alignments were performed with MAFFT, and phylogenetic trees were reconstructed using the maximum likelihood method implemented in IQ‐TREE. IQ‐TREE was executed using the ModelFinder Plus option (−m MFP) to identify the best‐fit substitution model, which was inferred to be Q.PLANT+F+R4. Branch support was assessed using 1000 bootstrap replicates.

### 2.6 Protein Extraction and DIA Analysis

Total proteins were extracted from a pool of 26 adult insects as previously described (Abbà et al. 2025). Data were acquired in data‐independent acquisition (DIA) mode following a previously described method (Abbà et al. 2025), with minor modifications. Full MS scans were acquired over an m/z range of 300–950 at a resolution of 70,000, with an AGC target of 1 × 10^6^ and a maximum injection time (IT) of 60 ms. DIA MS2 scans were acquired at a resolution of 17,500. The DIA method consisted of 22 isolation windows with an AGC target of 1 × 10^6^ and a maximum IT of 100 ms, including 5 windows of 31 m/z, 13 windows of 21 m/z (AGC target 2 × 10^5^ and maximum IT of 80 ms) and 4 windows of 51 m/z.

DIA raw files were analysed using DIA‐NN (version 2.2.0) with a spectral library generated from a custom proteome database. An FDR of 1% was applied at both the precursor and protein group levels. Proteins were digested in silico using trypsin with up to one missed cleavage allowed. Peptides ranging from 7 to 30 amino acids in length and carrying charge states of +1 to +4, and within a precursor m/z range of 275–975 were considered. Carbamidomethylation of cysteine residues was set as a fixed modification. An in silico spectral library was generated using deep learning–based prediction of MS/MS spectra and peptide retention times. Data analysis was performed using the ‘Generic’ scoring function. Protein inference was conducted in the ‘Protein names (from FASTA)’ mode with neural network–based, cross‐validated machine learning enabled.

### 2.7 Literature Searches

A systematic literature review on the occurrence of YLSs in insects was conducted by searching published literature with the following string (‘insect’ OR ‘insects’) AND (‘yeast‐like symbiont’ OR ‘obligate fungal symbionts’ OR ‘Ophiocordyceps‐allied’ OR ‘yeast‐like fungal symbionts’ OR ‘yeast‐like symbionts’ OR ‘yeast‐like microorganisms’ OR ‘Ophiocordyceps fungi’) in the scientific citation databases PubMed, Web of Science and Scopus during 2025. A critical step was the manual exclusion of true yeasts or YLSs, such as *Symbiotaphrina*, which belong to phylogenetically distinct fungal lineages from *Ophiocordyceps*‐allied fungi.

### 2.8 Identification of Potential Novel Insect Species Harbouring YLSs by a Two‐Step Bioinformatic Workflow

Sequence Read Archive (SRA) libraries to be analysed were selected from the NCBI (last accession March 2025) based on the following four criteria: (i) taxonomic classification within the orders Lepidoptera, Hemiptera, Diptera, Hymenoptera and Coleoptera, since they account for over 90% of the known terrestrial insect species (Stork 2018); (ii) RNA as the nucleic acid type, due to the smaller data size and ease of computational handling of RNA libraries compared to DNA libraries; (iii) Illumina as the sequencing platform, due to the high read quality and broad compatibility with standard tools for downstream processing; (iv) Europe as the geographic origin of the insect samples, to ensure the computational manageability of the dataset. Only for the order Hemiptera did we extend the survey to all publicly available transcriptomic SRA datasets, as, according to the literature, Ophiocordyceps‐allied yeast‐like symbionts are most likely to occur in this insect group.

SRA accession numbers and associated metadata were retrieved using the NCBI SRA Run Selector, applying the criteria described above. To identify insect species potentially associated with YLSs, SRA reads were aligned against the coding sequences of 18 ribosomal proteins and cytochrome c oxidase subunit I (COI) previously identified in StYLS through metatranscriptomic analyses (Abbà et al. 2022) (Data S2). A custom Bash script automated Magic‐BLAST v1.7.0 (−reftype transcriptome) (Boratyn et al. 2019) read mapping for the list of selected SRA accessions, generating a table of mapped read counts for each accession.

Libraries with ≥ 20 mapped reads were downloaded for further analysis. If multiple libraries of the same species were above the threshold, one library was selected per species based on the highest number of matching reads. Illumina reads were subjected to quality control using the BBTools suite and assembled using SPAdes v4.2.0 in ‘meta’ mode with default parameters. The resulting transcripts were queried against the NCBI core‐nr database using DIAMOND BLASTx to identify fungal transcripts belonging to genera within the family Ophiocordycipitaceae, according to NCBI Taxonomy.

All bioinformatic analyses were conducted on a high‐performance workstation with a 16‐core CPU, 64 GB of RAM and a 1 TB NVMe SSD running Ubuntu 24.04. An additional 4 TB HDD was used to store datasets and intermediate files.

## 3 Results

### 3.1 Genome Sequencing of StYLS Reveals the Retention of Metabolic Pathways Beyond Essential Nutrient Biosynthesis

**FIGURE 1:**
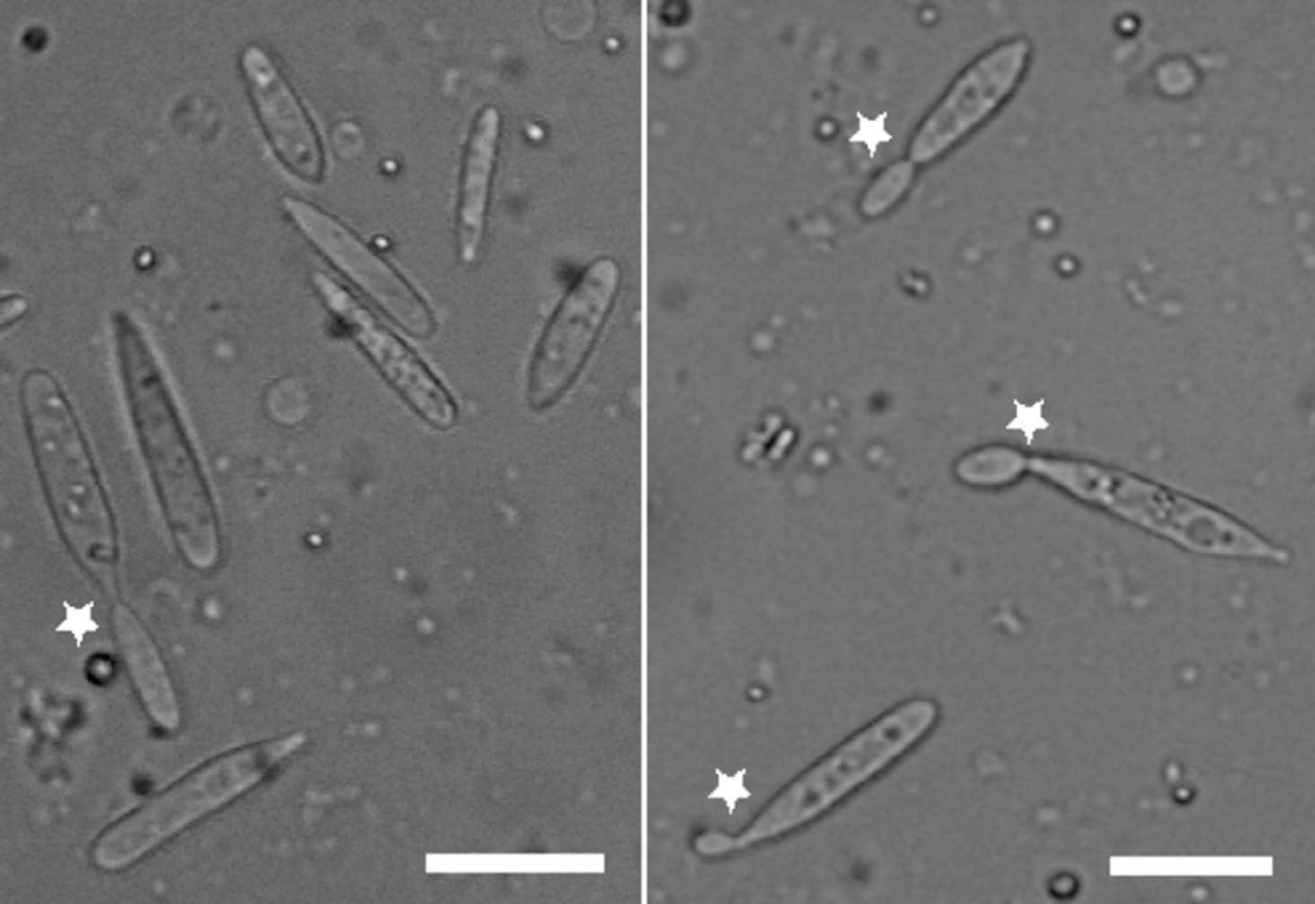
Yeast‐like symbionts (YLS) in *Scaphoideus titanus* fat body homogenates. StYLS cells observed under an optical microscope exhibit an elongated, fusiform morphology. Stars indicate budding cells. Magnification bar corresponds to 10 μm.

To assess the enrichment of yeast‐like symbionts (YLS) in *S. titanus*, fat body homogenates were examined microscopically. Numerous elongated fusiform cells were observed with dimensions ranging from 8.34 to 19.05 μm in length (mean value 13.29 μm ± 2.45 μm, *n* = 95) and from 1.71 to 3.54 μm in width (mean value 2.77 μm ± 0.35 μm, *n* = 95). Many cells had one rounded and one pointed end, and some of them exhibited yeast‐like budding (Figure 1).

Despite repeated attempts, StYLS could not be isolated in vitro, as is typical for most YLSs, with the only exception of the YLS isolated from the cicada *Meimuna opalifera* (Matsuura et al. 2018). Consequently, DNA was extracted from the abdomens of *S. titanus* adults to increase the likelihood of sequencing StYLS, which has previously been localized in the fat bodies (Sacchi et al. 2008).

Analysis of the StYLS genome (217.13 Mbp and 7605 protein‐coding genes) using BUSCO revealed a completeness score of 94.3%, with 2.2% of genes fragmented and 3.5% missing. Consequently, we restricted downstream analyses only to features that were present, as missing genes may reflect assembly incompleteness rather than true absence.

**FIGURE 2:**
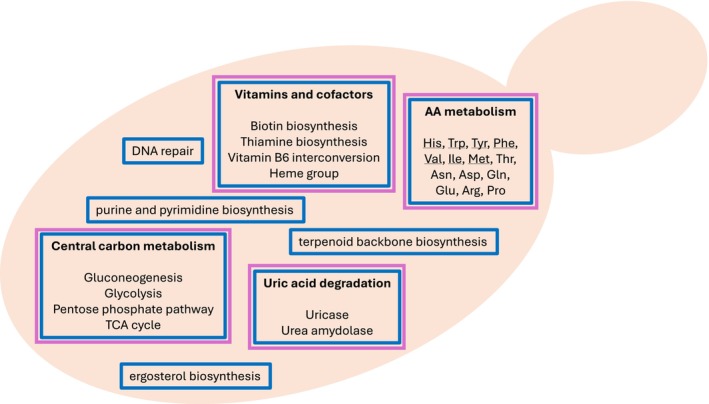
Metabolic pathways detected in StYLS genome using KEGG Automatic Annotation Server and BlastKOALA. Blue rectangles indicate complete pathways, while pink ones indicate pathways for which one or more proteins were detected by Data‐Independent Acquisition (DIA) proteomic analysis. AA: amino acid. Amino acids that are essential for insect survival are underlined.

According to KEGG annotation (Figure 2), the StYLS genome encodes complete pathways for central carbon metabolisms, including glycolysis, gluconeogenesis, the TCA cycle, and the pentose phosphate pathway, as well as de novo purine and pyrimidine biosynthesis. Complete biosynthetic pathways were identified for several amino acids essential for the insect host (i.e., tryptophan, tyrosine, phenylalanine, valine, isoleucine, methionine, histidine and threonine), as well as for non‐essential ones (i.e., aspartate, asparagine, glutamate, glutamine, proline and arginine). Additionally, the metabolic reconstruction showed complete pathways for the biosynthesis of biotin and thiamine, heme production and the synthesis of ergosterol and terpenoid backbones. Notably, all the enzymes that lead to the conversion of uric acid to ammonia were also present (Data S3 and S4). Surprisingly for an obligate endosymbiont, the core DNA repair machinery appeared to be fully retained. Unlike the YLS associated with *Parthenolecanium corni* (Ward et al. 2024), StYLS retains genes encoding proteins containing a heat‐labile enterotoxin domain, some of which possess a signal peptide, and several MAP kinases related to hyphal growth and/or cell wall integrity, such as WSC, ROM1_2, RHOA, PRKCA, MKK1_2, MAPK7, STE12 (Cong et al. 2022; Gómez‐Gil et al. 2021; Zhao et al. 2007).

**FIGURE 3:**
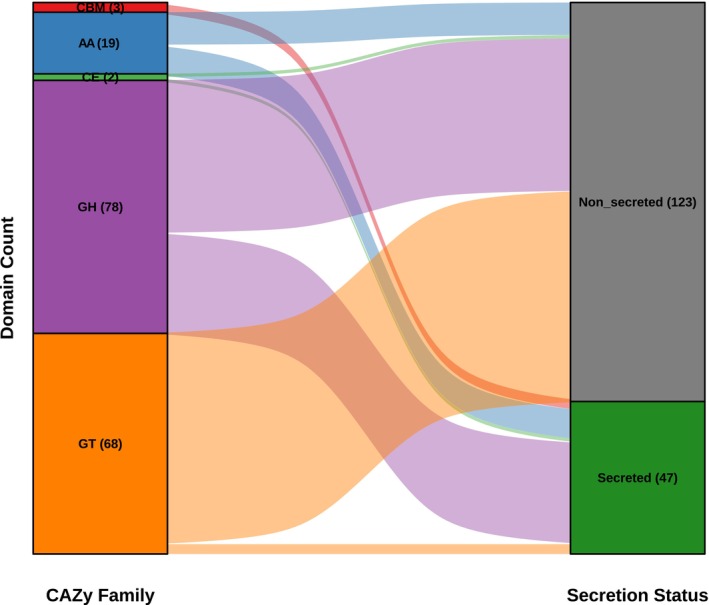
Distribution of CAZy families by predicted secretion status. The alluvial plot illustrates the flow from CAZy families (left) to their respective secretion profiles predicted by SignalP (right). Data are derived from Data S5. The width of each coloured ‘edge’ (alluvium) is proportional to the total domain count indicated by values in parentheses. Counts represent every individual domain hit found by HMMER, including those separated by ‘+’ signs within the same sequence. The CAZy families represented in this figure include glycoside hydrolases (GH), responsible for hydrolyzing glycosidic bonds; carbohydrate‐binding modules (CBM), which mediate binding to carbohydrate substrates; glycosyl transferases (GT), enzymes that catalyse the transfer of sugar moieties; auxiliary activities (AA), which include redox enzymes that assist in polysaccharide degradation; and carbohydrate esterases (CE), which remove ester‐based modifications from carbohydrates.

The CAZyme profile comprised a diverse set of enzymes involved in the synthesis and degradation of complex carbohydrates (Figure 3, Data S5). A total of 161 proteins were identified as containing at least one CAZy domain, representing 68 distinct CAZy HMMER domains distributed across three major functional families: glycoside hydrolases (GH), glycosyltransferases (GT) and auxiliary activities (AA). The CAZyme repertoire was dominated by the β‐glycan synthase family GT2 (*n* = 16), which primarily comprises chitin synthases, followed by the chitinase‐associated family GH18 (*n* = 10) and the oxidoreductase family AA3 (*n* = 8). SignalP‐based secretome analysis predicted extracellular localization for 30 glycoside hydrolases and 9 oxidoreductases, all likely involved in extracellular polysaccharide or glycoprotein modification.

### 3.2 Amino Acid Metabolism, Protein Folding and Nitrogen Recycling Are the Main Functions of Fungal Proteins Expressed in the Insect

A Data‐Independent Acquisition (DIA) proteomic analysis was employed to experimentally verify which proteins are actually expressed by StYLS within the insect host reared under controlled laboratory conditions. The sensitivity of this approach for detecting low‐abundance peptides is critical for identifying fungal proteins that may be masked by the higher abundance of host insect proteins.

As *S. titanus* does not have a fully sequenced genome, the predicted proteins derived from previously assembled *S. titanus* transcripts were supplemented with proteomic datasets from the phylogenetically close leafhoppers *Macrosteles quadrilineatus* (accession GCF_028750875.1) and *Euscelidius variegatus* (accession GCA_053769955.1), as described in (Abbà et al. 2025). Based on prior knowledge of the presence of the primary symbiont *Karelsulcia* and the secondary symbiont *Cardinium* (only in European populations) within the *S. titanus* microbiome, the deduced proteome of the newly sequenced StYLS was combined with the predicted proteins derived from: (i) the merged transcriptome assemblies of *S. titanus* (BioProject PRJNA765507), (ii) ‘*Candidatus* Karelsulcia muelleri’ strain Eva_TO (endosymbiont of *E. variegatus*, accession CP142806) and Cardinium endosymbiont of *Sogatella furcifera* (Genome Assembly GCF_003351905.1). These two bacterial symbionts were selected because their insect hosts are phylogenetically closely related to *S. titanus*, making them suitable for identifying potential symbiont‐derived proteins in our dataset.

A total of 1513 distinct protein groups were identified using DIA‐NN (Data S6). Following taxonomic filtering, 27 of these groups were ascribed to StYLS. After manual curation to resolve redundancy and group overlaps, 25 unique fungal protein identifications were retained for functional analysis (Table 1). Of these, six were related to amino acid biosynthesis, including two involved in methionine metabolism, that is, cobalamin‐independent methionine synthase (MetE) and S‐adenosylhomocysteine hydrolase (AHCY).

**TABLE 1:** Functional classification of unique StYLS proteins identified by proteomic analysis.

Functional category	Accession	Annotation	Organism
Uric acid degradation	XP_044717643.1	Uricase domain‐containing protein	*Hirsutella rhossiliensis*
	KAF4511964.1	Urea amidolyase (Urea carboxylase/Allophanate hydrolase)	*Ophiocordyceps sinensis*
Amino acid metabolism	XP_044718292.1	Cobalamin‐independent methionine synthase (MetE)	*Hirsutella rhossiliensis*
	XP_044723945.1	Cys/Met metabolism PLP‐dependent enzyme	*Hirsutella rhossiliensis*
	XP_044716946.1	Acetohydroxy acid isomeroreductase	*Hirsutella rhossiliensis*
	KJZ73038.1	Adenosylhomocysteinase (AHCY)	*Hirsutella minnesotensis*
	XP_044721624.1	Semialdehyde dehydrogenase domain‐containing protein	*Hirsutella rhossiliensis*
	PHH81514.1	Serine hydroxymethyltransferase (cytosolic)	*Cordyceps* sp.
Central carbon metabolism	KJZ75863.1	Transaldolase	*Hirsutella minnesotensis*
	XP_044714913.1	hpcH/HpaI aldolase/citrate lyase family protein	*Hirsutella rhossiliensis*
Energy metabolism	XP_044724832.1	ATP synthase alpha/beta family protein	*Hirsutella rhossiliensis*
	RGP73302.1	Peptidyl‐prolyl cis‐trans isomerase, mitochondrial	*Fusarium sporotrichioides*
	PFH59638.1	ADP/ATP transporter (Adenylate translocase)	*Ophiocordyceps unilateralis*
Oxidative stress	XP_044718654.1	Peroxidase domain‐containing protein	*Hirsutella rhossiliensis*
Proteostasis	XP_044721175.1	Hsp90 protein	*Hirsutella rhossiliensis*
	XP_044721992.1	Hsp70 protein	*Hirsutella rhossiliensis*
	XP_044724469.1	Hsp70 protein	*Hirsutella rhossiliensis*
	XP_044714992.1	Hsp20/alpha‐crystallin family protein	*Hirsutella rhossiliensis*
	XP_044724580.1	TCP‐1/cpn60 chaperonin family protein	*Hirsutella rhossiliensis*
Signalling and structure	EQUATION L04267.1	Ran GTPase	*Ophiocordyceps sinensis*
	KAF4511485.1	CFEM domain‐containing protein	*Ophiocordyceps sinensis*
	XP_044722260.1	2OG‐Fe(II) oxygenase superfamily protein	*Hirsutella rhossiliensis*
Vitamins and cofactors biosynthesis	XP_044720270.1	SOR/SNZ family protein (Vitamin B6 biosynthesis)	*Hirsutella rhossiliensis*
Hypothetical/uncharacterized	KFA64977.1	Hypothetical protein S40285_10147	*Stachybotrys chlorohalonatus*
	KJZ78419.1	Hypothetical protein HIM_02457	*Hirsutella minnesotensis*

*Note:* The table lists identified proteins by their functional role, including conserved domain descriptions and NCBI accession numbers.

Several proteins were associated with protein homeostasis and stress defence, such as heat shock proteins, chaperones and a peroxidase domain‐containing protein. Additionally, uricase and urea amidolyase were detected, which are involved in the conversion of uric acid into ammonia, a key step in nitrogen recycling.

### 3.3 Proteome‐Wide Phylogeny Indicates a Relationship Between StYLS and the Genera *Ophiocordyceps* and *Hirsutella*

**FIGURE 4:**
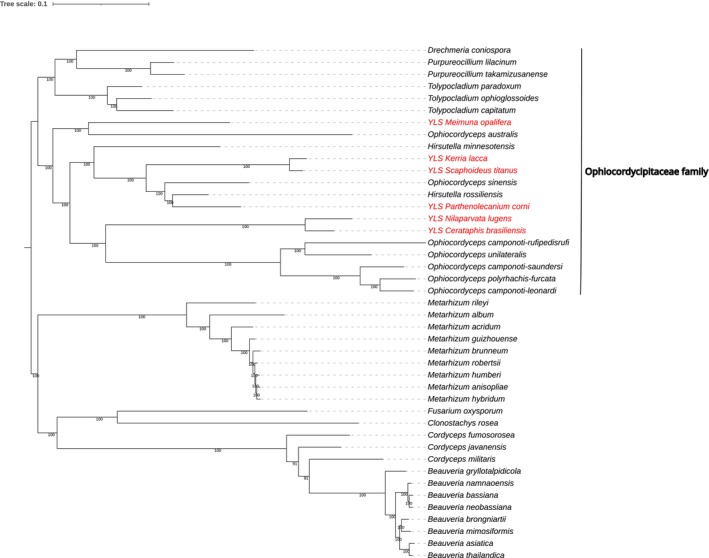
Phylogenomic placement of StYLS within Hypocreales. Hypocreales core proteins were identified in StYLS, in five previously sequenced yeast‐like symbiont genomes (*Meimuna opalifera*, *Kerria lacca*, *Parthenolecanium corni*, *Nilaparvata lugens* , and *Cerataphis brasiliensis*), and in 37 additional genomes representing species of Ophiocordycipitaceae and other members of Hypocreales (Data S1). Phylogenetic relationships were inferred using PhyloPhlAn v3 with MAFFT alignments and maximum likelihood tree reconstruction in IQ‐TREE (ModelFinder best‐fit model Q.PLANT+F+R4), with branch support estimated from 1000 bootstrap replicates. Yeast‐like symbiont taxa are indicated in red.

To resolve the evolutionary position of the StYLS genome, we performed a core protein prediction using BUSCO across the other five available YLS genomes, that is, the YLSs of *M. opalifera* (Matsuura et al. 2018), *Kerria lacca* (Vaishally et al. 2025), *Pa. corni* (Ward et al. 2024), *Nilaparvata lugens* (Fan et al. 2015) and *Cerataphis brasiliensis* (Vogel and Moran 2013). These were compared with reference core proteins identified in genera belonging to the *Ophiocordycipitaceae* family, as well as in several other genera known to include entomopathogens in the order Hypocreales (e.g., *Metarhizium*, *Beauveria* and *Purpureocillium*). The final phylogenomic analysis was based on a concatenated alignment of core proteins across 43 genomes (Data S1), totalling more than 1.3 million amino acid sites (Figure 4).

StYLS appears most closely related to the YLS associated with *K. lacca*, both of which were part of a well‐defined clade that includes *Ophiocordyceps sinensis* and two species of *Hirsutella*, that is, *H. rhossiliensis* and *H. minnesotensis* . Interestingly, the YLS from *Pa. corni* falls within the same cluster but shows a closer phylogenetic affinity to *O. sinensis* and *H. rhossiliensis* than to the other two YLSs. YLSs from *N. lugens* and *C. brasiliensis* are sister taxa that cluster with a group of *Ophiocordyceps* species distinct from the one that included the previous three YLSs. In contrast, the YLS from *M. opalifera* branches with *Ophiocordyceps australis* forming a distinct branch from the one that includes all the other YLSs. In conclusion, the six YLS genomes are more closely related to *Hirsutella* and *Ophiocordyceps* species than to any other member of the order *Hypocreales* included in the analysis.

### 3.4 Literature‐Based Overview of Known Insect–YLS Associations

The potential monophyletic origin of yeast‐like symbionts established an evolutionary foundation that significantly enhances the reliability of a sequence‐similarity search for the identification of new candidate YLS–insect associations through a newly developed bioinformatic pipeline. Initially, we conducted a literature survey to provide a comprehensive overview of the insect species reported to harbour yeast‐like symbionts. This curated list served as a reference set for defining positive‐control species in the global bioinformatic analyses.

According to our survey, at least 160 hemipteran species have been reported in the literature as being associated with YLSs (Data S7). Records in which insect hosts were reported only generically (e.g., ‘unknown planthoppers’) or identified only at the family or subfamily level were excluded from the species count to ensure taxonomic precision.

### 3.5 In Silico Analysis of SRA RNA Libraries Revealed Marked Variation in YLS‐Mapped Read Abundance Across Libraries of the Same Species

Based on our literature survey, we first examined transcriptomic SRA datasets from two known YLS‐associated hemipterans, *N. lugens* and *Ericerus pela*, both represented by multiple datasets (Data S8 and S9). This preliminary analysis assessed how effectively our newly developed pipeline detects YLS‐associated insects and whether a single library per species is adequate for reliable detection.

We therefore compared the abundance of YLS‐mapped reads across libraries representing different tissues and developmental stages. Fat bodies in *N. lugens* libraries showed the highest number of YLS‐mapped reads, which is consistent with their known role as the primary tissue harbouring YLSs in this species. In contrast, the number of mapped reads varied markedly across other tissues and organs and was nearly absent in salivary glands and head samples.

Evaluation of YLS‐mapped reads in *E. pela* libraries revealed substantial variation also across life stages and among biological replicates of the same stage. These differences likely reflect variation in YLS transcriptional activity (i.e., low abundance but high transcriptional activity), variation in YLS abundance within the host (i.e., high abundance but low transcriptional activity) or a combination of both.

Given the variability of these results, we analysed all available libraries for each species to maximize the chance of identifying new YLS hosts. Additionally, this variability suggested caution when defining a threshold number of mapped reads for identifying a species as a potential YLS host, given that the number of hits in *N. lugens* ranged from 0 to 1711, and in *E. pela* from 9 to over 30,000. Nonetheless, the large volume of data involved in broad SRA screening required the application of a threshold, which may occasionally lead to false‐negative results. Libraries were considered potential candidates for further analysis if they met a minimum threshold of 20 aligned reads, ideally corresponding to at least one read per target YLS transcript.

### 3.6 A Broad SRA Screening Identifies Potentially Unreported Insect–YLS Associations

Following this initial assessment, we expanded our analysis to a comprehensive survey of RNA SRA datasets across multiple insect species. Although all insect hosts reported to date to harbour YLSs belong exclusively to the order Hemiptera (Data S7), we broadened our in silico screening to other insect orders to test whether their host range might extend beyond this group.

The number of SRA accessions analysed for each insect order, along with the total number of species, is summarized in Table 2 and provided in full in Data S10. The accessions with at least 20 mapping reads corresponded to 144 distinct insect species, including 123 hemipteran species, eight lepidopteran species, one hymenopteran species, two dipteran species and ten coleopteran species.

**TABLE 2:** Summary of the meta‐analysis results for the presence of *Ophiocordyceps*‐allied yeast‐like symbionts (YLS) across five insect orders after a two‐step refinement process.

Order	Number of analysed species	Number of positive insect species after the first step	Number of positive insect species after the second step
Coleoptera	246 (909)	10 (12)	1
Diptera	110 (569)	2 (10)	0
Hymenoptera	210 (647)	1 (1)	1
Lepidoptera	359 (1690)	8 (30)	0
Hemiptera	776 (4291)	123 (386)	56
*Total*	*1701*	*144*	*58*

*Note:* Number of positive SRA accessions are reported in brackets. The geographic restriction to Europe was applied to ensure the manageability of the dataset. However, for the order Hemiptera, we extended the survey to include all publicly available transcriptomic SRA datasets, as the literature indicates that this order comprises the hosts in which *Ophiocordyceps*‐allied yeast‐like symbionts are most likely to occur.

We then compared the results of the literature survey with the bioinformatics results to check the accuracy of the latter. Of the 22 YLS‐bearing species reported in the literature (Data S7 and S10) for which SRA accessions meeting the selection criteria are publicly available, 15 were positive in the bioinformatics analysis. The exceptions were the cicada *Meimuna mongolica*; the planthoppers *Laodelphax striatellus* , *Geisha distinctissima*, *Acanalonia conica* and *Ricania speculum*; and the soft scale insects *Ceroplastes floridensis* and *Coccus hesperidum* . Additionally, the bioinformatics analysis revealed 129 potential new host species, 108 of which are hemipterans. Accession ERR10751845 was excluded from further analyses because the insect from which the library was derived is generically reported as a hemipteran. Only insects identified at least to the genus level were retained. Among all negative controls known not to harbour YLSs (Kobiałka, Michalik, Szwedo, and Szklarzewicz 2018; Koga and Moran 2014), only library SRR7943171 from *Nephotettix cincticeps* tested positive after this initial screening step.

Following this preliminary screening, a more detailed and computationally demanding analysis was performed to improve the identification of potential YLSs, as alignments to ribosomal and COI transcripts may also recover fungi serendipitously associated with insects, such as surface contaminants, pathogens or transiently present fungi in the insect midgut. Therefore, for each positive insect species, only the SRA library with the highest hit count was assembled and analysed by BLASTx against the NCBI nr database to search for transcripts with significant similarity to proteins belonging to the Ophiocordycipitaceae family.

The threshold for the minimum number of YLS‐assigned transcripts required to identify an insect as a potential YLS‐bearing host was established using a library from a confirmed YLS‐bearing insect, *Hishimonus phycitis* (SRR16560730), which exhibited the lowest number of transcripts assigned to Ophiocordycipitaceae (23 transcripts) among the 15 positive controls. The library from the negative control *Nephotettix cincticeps* that was positive after the initial screening step was removed at this stage, as no transcripts were assigned to any members of the Ophiocordycipitaceae. A total of 58 assemblies showed a number of *Ophiocordycipitaceae*‐related transcripts equal to or exceeding the predefined threshold (Data S10). Fifteen of these corresponded to the positive controls, while the remaining 43 represent potential novel YLS‐bearing species (Table 3). All of these were hemipterans, with only two exceptions: *Leptochilus alpestris* (Hymenoptera) and *Lydus trimaculatus* (Coleoptera). Our findings represent the first record of potential YLS‐associated insects in some hemipteran lineages previously not known to harbour such symbioses. Specifically, this includes members of the family Eriococcidae as well as the family Membracidae. Additionally, our results provide the first evidence of putative YLS‐bearing insects within the suborder Heteroptera, represented by the families Gerridae, Hydrometridae and Dinidoridae. Therefore, all known YLS‐associated insect species, as well as all potential new ones, belong to three of the four hemipteran suborders, that is, Auchenorrhyncha, Sternorrhyncha and Heteroptera. In our analysis, no potential YLS‐bearing species was identified among the six Coleorrhyncha libraries available in the SRA database (*Hackeriella veitchi*, *Oiophysa cumberi*, *Peloridium pomponorum*, *Xenophyes cascus*, *X. metoponcus* and *Xenophysella greensladeae*).

**TABLE 3:** Potential novel YLS‐bearing species obtained from the SRA survey.

Species	Order	Suborder	Family
*Lydus trimaculatus*	Coleoptera	Polyphaga	Meloidae
*Acanthocasuarina muellerianae*	Hemiptera	Sternorrhyncha	Triozidae
*Acanthococcus lagerstroemiae*	Hemiptera	Sternorrhyncha	Eriococcidae
*Aetalion reticulatum*	Hemiptera	Auchenorrhyncha	Aetalionidae
*Athysanopsis salicis*	Hemiptera	Auchenorrhyncha	Cicadellidae
*Austrotartessus* sp.	Hemiptera	Auchenorrhyncha	Cicadellidae
*Balala* sp. *YHH‐2021*	Hemiptera	Auchenorrhyncha	Cicadellidae
*Batracomorphus* sp. *YHH‐2021*	Hemiptera	Auchenorrhyncha	Cicadellidae
*Bhatia hastata*	Hemiptera	Auchenorrhyncha	Cicadellidae
*Coccus* sp. *AD‐2014*	Hemiptera	Sternorrhyncha	Coccidae
*Cosmococcus erythrinae*	Hemiptera	Sternorrhyncha	Eriococcidae
*Destinoides conspicuus*	Hemiptera	Auchenorrhyncha	Cicadellidae
*Drabescus henanensis*	Hemiptera	Auchenorrhyncha	Cicadellidae
*Eusarima* sp.	Hemiptera	Auchenorrhyncha	Issidae
*Frequenamia cavifrons*	Hemiptera	Auchenorrhyncha	Cicadellidae
*Graphocephala coccinea*	Hemiptera	Auchenorrhyncha	Cicadellidae
*Hishimonoides sellatiformis*	Hemiptera	Auchenorrhyncha	Cicadellidae
*Hishimonus sellatus*	Hemiptera	Auchenorrhyncha	Cicadellidae
*Hydrometra stagnorum*	Hemiptera	Heteroptera	Hydrometridae
*Kerria chinensis*	Hemiptera	Sternorrhyncha	Kerriidae
*Ladella* sp. *AD‐2014*	Hemiptera	Auchenorrhyncha	Cicadellidae
*Ledra* sp. *YHH‐2021*	Hemiptera	Auchenorrhyncha	Cicadellidae
*Lycoderes burmeisteri*	Hemiptera	Auchenorrhyncha	Membracidae
*Macropsis matsumurana*	Hemiptera	Auchenorrhyncha	Cicadellidae
*Megymenum gracilicorne*	Hemiptera	Heteroptera	Dinidoridae
*Metopolophium dirhodum*	Hemiptera	Sternorrhyncha	Aphididae
*Micrutalis calva*	Hemiptera	Auchenorrhyncha	Membracidae
*Mileewa jianzhuensis*	Hemiptera	Auchenorrhyncha	Cicadellidae
*Neogerris hesione*	Hemiptera	Heteroptera	Gerridae
*Nephotettix nigropictus*	Hemiptera	Auchenorrhyncha	Cicadellidae
*Nilaparvata muiri*	Hemiptera	Auchenorrhyncha	Delphacidae
*Nionia palmeri*	Hemiptera	Auchenorrhyncha	Cicadellidae
*Olidiana hamularis*	Hemiptera	Auchenorrhyncha	Cicadellidae
*Paradorydium reflexanum*	Hemiptera	Auchenorrhyncha	Cicadellidae
*Paratachardina pseudolobata*	Hemiptera	Sternorrhyncha	Kerriidae
*Phlogotettix cyclops*	Hemiptera	Auchenorrhyncha	Cicadellidae
*Phlogothamnus fanjingshanensis*	Hemiptera	Auchenorrhyncha	Cicadellidae
*Recilia dorsalis*	Hemiptera	Auchenorrhyncha	Cicadellidae
*Ricania simulans*	Hemiptera	Auchenorrhyncha	Ricaniidae
*Schlechtendalia chinensis*	Hemiptera	Sternorrhyncha	Aphididae
*Stenotortor* sp. *YHH‐2021*	Hemiptera	Auchenorrhyncha	Cicadellidae
*Truncatocornum parvum*	Hemiptera	Auchenorrhyncha	Membracidae
*Leptochilus alpestris*	Hymenoptera	Apocrita	Vespidae

Across all confirmed and newly identified candidate YLS‐associated hemipterans in our dataset, nearly all species belong to sap‐feeding lineages, either phloem‐ or xylem‐feeders (Table 4). The only exceptions to this trophic pattern are the two heteropteran species *Neogerris hesione* and *Hydrometra stagnorum* , which are predators or scavengers.

**TABLE 4:** Taxonomic distribution and feeding strategies of the YLS‐associated insects.

Suborder	Suborder count	Family	Family count	Feeding habit
Auchenorrhyncha	144	Cicadidae	79	Sap‐sucking
		Cicadellidae	35	Sap‐sucking
		Delphacidae	12	Sap‐sucking
		Ricaniidae	5	Sap‐sucking
		Flatidae	4	Sap‐sucking
		Aetalionidae	2	Sap‐sucking
		Issidae	2	Sap‐sucking
		Membracidae	3	Sap‐sucking
		Acanaloniidae	1	Sap‐sucking
		Dictyopharidae	1	Sap‐sucking
Sternorrhyncha	54	Coccidae	39	Sap‐sucking
		Aphididae	8	Sap‐sucking
		Kerriidae	3	Sap‐sucking
		Eriococcidae	2	Sap‐sucking
		Kermesidae	1	Sap‐sucking
		Triozidae	1	Sap‐sucking
Heteroptera	3	Dinidoridae	1	Sap‐sucking
		Gerridae	1	Predatory/scavenger
		Hydrometridae	1	Predatory

*Note:* The table reports the summary of the taxonomic classification of known and newly identified candidate YLS‐associated insects, along with their corresponding frequencies. The table also classifies them based on the predominant feeding habits of their respective insect families.

## 4 Discussion

### 4.1 The Metabolic Potential of StYLS and Its Functional Impact on Its Insect Host

Yeast‐like symbionts play a vital role in insect nutrition by supplying essential amino acids, vitamins, cofactors and sterol precursors, and by recycling nitrogenous waste into bioavailable forms (Noda and Koizumi 2003; Matsuura et al. 2018; Xue et al. 2014; Hongoh and Ishikawa 2000). Such intimate symbiotic partnerships can also confer novel metabolic capabilities, including the biosynthesis of lac pigments (Vaishally et al. 2025).

The leafhoppers *E. variegatus* and *M. quadrilineatus* , though phylogenetically close to *S. titanus*, rely on two bacterial endosymbionts, ‘*Candidatus* Karelsulcia muelleri’ and ‘*Candidatus* Nasuia deltocephalinicola’ (*Pseudomonadota*, hereafter *Nasuia*) (Abbà et al. 2025; Bennett and Moran 2013; Cooper et al. 2023). In *S. titanus*, where only *Karelsulcia* is present, the genome of StYLS reveals a high degree of metabolic autonomy, in sharp contrast to the pronounced genome reduction characterising ancient obligate bacterial endosymbionts like *Nasuia* (< 120 kbp). StYLS, in fact, retains central carbon metabolic pathways, DNA repair mechanisms and a broad repertoire of CAZymes, which are likely involved in fungal cell wall synthesis and remodelling. Notably, it possesses complete biosynthetic pathways for both methionine and histidine, providing the two essential amino acids not synthesised by *Karelsulcia* (McCutcheon and Moran 2010), while redundantly supplementing the host with a broader suite of additional essential amino acids (i.e., threonine, valine, isoleucine, phenylalanine, tryptophan and arginine) usually provided by this bacterial endosymbiont. Although evidence of genome degradation and intensified positive selection were observed in the YLSs of *Parthenolecanium corni* (Ward et al. 2024) *Nilaparvata lugens* (Fan et al. 2015) and *Cerataphis brasiliensis* (Vogel and Moran 2013), the relatively recent origin of these associations, compared with ancient bacterial endosymbionts, may explain the absence of extensive gene loss in these endosymbiotic fungi, as observed in StYLS.

The genome annotation of StYLS, alongside the proteins identified by the DIA approach, indicates that StYLS possesses the metabolic capacity to degrade uric acid and convert it into reusable nitrogen. Although insects are primarily uricotelic (Weihrauch and O'Donnell 2021), excreting nitrogen as uric acid via the Malpighian tubules, StYLS may additionally enable the storage and reutilization of uric acid as a nitrogen reserve, as demonstrated for the yeast‐like symbiont of *N. lugens* (Sasaki et al. 1996; Hongoh and Ishikawa 2000).

While *E. variegatus* and *M. quadrilineatus* primarily feed on sap of a broad range of plants, *S. titanus* is quite a strict monophagous that feeds almost exclusively on the genus *Vitis* (grapevine) and depends on it to complete its biological cycle. The acquisition of StYLS might have enabled the exploitation of this highly specialized diet (Cornwallis et al. 2023) not only by providing the missing nutrients, as suggested by the expression of proteins involved in amino acid biosynthesis and nitrogen recycling, but also by potentially mitigating the physiological challenges of feeding on grapevine phloem. In particular, StYLS may counteract the toxic effects of reactive oxygen species generated by the plant phenolic compounds and quinones through the activity of stress‐ and proteostasis‐related proteins identified in our analysis.

### 4.2 The Phylogeny of YLSs Does Not Mirror That of Their Insect Hosts

These differences in microbiome composition among phylogenetically close insects further support the conclusion that the evolutionary history of these fungal symbionts is largely incongruent with the evolutionary history of their hosts (Gomez‐Polo et al. 2017; Wang et al. 2022). Additionally, we showed that the StYLS proteome shares greater similarity with the YLSs of the scale insects *K. lacca* and *Pa. cornii* than with that of the brown planthopper *N. lugens* . Instead, the latter appears more closely related to the YLS of the aphid *C. brasiliensis* . This relationship is noteworthy when considered in the context of host phylogeny: planthoppers and leafhoppers belong to Auchenorrhyncha, whereas scale insects and aphids are part of the phylogenetically distinct hemipteran suborder Sternorrhyncha. Incongruence between the phylogeny of insect hosts and that of their YLSs has been observed particularly in cicadas and scale insects, where analyses based on ribosomal RNA sequences or mitogenomes from multiple species have revealed a complex history of repeated acquisitions, losses, and replacements of this fungus (Zhou et al. 2025; Gomez‐Polo et al. 2017; Matsuura et al. 2018; Vashishtha et al. 2011; Podsiadło et al. 2018).

All six YLS proteomes analysed in this work, including the one from the cicada *M. opalifera* (the only YLS that has so far been cultured), form a supported monophyletic clade with species of *Hirsutella* and *Ophiocordyceps*. These two genera are widely regarded as a single evolutionary unit, representing the sexual and asexual stages, respectively, of the same fungal lineage. Although the phylogenetic tree also included other genera of Ophiocordycipitaceae and, more broadly, of Hypocreales, the deduced proteomes of all YLS genomes sequenced to date cluster exclusively with *Hirsutella* and *Ophiocordyceps*. This is consistent with previous phylogenetic studies that independently identified the YLSs associated with *M. opalifera* and *Pa. corni* as close relatives of *Ophiocordyceps* (Zhou et al. 2025; Matsuura et al. 2018). By expanding the phylogenetic analysis to include the proteomes of six sequenced YLSs across diverse insect lineages, our results provide support for the theory that the genus *Ophiocordyceps* may represent the evolutionary origin of these fungal endosymbionts. YLSs are thought to derive from ancestral entomopathogenic fungi, formerly classified within Clavicipitaceae, that transitioned from a lethal parasitic lifestyle to a mutualistic, intracellular association with their insect hosts (Suh et al. 2001). Species of free‐living *Ophiocordyceps* are often characterized by a high degree of host specialization and, in some cases, the ability to manipulate host behaviour (van Roosmalen and de Bekker 2024), traits that might have facilitated the evolutionary transition from pathogenicity to obligate endosymbiosis. The StYLS genome contains putative secreted glycoside hydrolases, oxidoreductases and heat‐labile enterotoxins, which may represent evolutionary remnants of the free‐living entomopathogenic ancestor. In free‐living *Ophiocordyceps* species, enterotoxins are specifically expressed during host behavioural manipulation (Kobmoo et al. 2018). In StYLS, glycoside hydrolases and oxidoreductases may be involved primarily in fungal cell wall remodelling, whereas enterotoxins may be associated with stress responses and the maintenance of cell‐surface carbohydrate profiles, as observed in *Beauveria bassiana* (Ding et al. 2023). Their predicted secretion, however, still makes them plausible mediators of host–symbiont interactions.

### 4.3 A Flexible Two‐Step Approach for High‐Accuracy SRA Mining for YLS‐Insect Associations

The polyphyletic origin of insects associated with YLSs prompted an assessment of the prevalence of such symbiosis. To address this, we examined the occurrence of *Ophiocordyceps*‐allied fungi in a broad range of insect species through transcriptomic SRA data mining combined with literature surveys.

As of September 2025, the NCBI SRA database contains more than 40 million records, with an annual growth rate of approximately 16% (Sayers et al. 2026). This vast and rapidly growing resource has created a new bioinformatic challenge: efficiently screening petabytes of sequencing data to identify sequences of interest. The Serratus project, for example, has demonstrated the power of SRA‐wide mining to identify tens of thousands of novel viral sequences (Edgar et al. 2022). Designed for more general‐purpose searches, Logan‐Search (Chikhi et al. 2024) uses a k‐mer index built from a massive assembly of SRA data and, allowing a single input sequence, rapidly identifies matching SRA accessions within minutes. Using our YLS sequences shorter than 1000 bp one by one, since longer sequences cannot be processed, we identified matches in 11 insect species, including only two positive controls, *S. titanus*, the original source of the YLS sequences, and *K. lacca*. However, literature evidence indicates that YLS–insect associations are more widespread. Similar results were obtained using another k‐mer‐based tool, MetaGraph (Karasikov et al. 2025), which allows a maximum of 10 query sequences per search. As these methods proved to be ineffective for our purposes, we developed an ad hoc pipeline. This two‐step workflow offers a balance between scalability and accuracy and can be adapted for different targets. Initially, it involved the selection of SRA datasets through the NCBI Run Selector, which allows filtering by species, library type, or other metadata criteria. The first step of our bioinformatic analysis used Magic‐BLAST, which queries the selected SRA datasets online against any number of target sequences of any length and performs full alignments in real‐time, without the need to download the selected datasets locally. In addition, it provides higher accuracy in detecting true matches compared to k‐mer‐based searches, even if streaming each dataset requires a longer runtime (an average of 32 min per library). This first step of our pipeline significantly reduced the number of candidate SRA datasets for subsequent analysis, which involved library downloading, assembly, and querying against a locally hosted NCBI nr database. Importantly, despite its low throughput, this approach does not require cloud computing, remains computationally manageable on a standard workstation, and can be easily adapted to different target sequences and libraries.

The first challenge in selecting SRA libraries to detect potential YLS‐bearing insects was the quality of the associated BioSample metadata, which are organized as structured ‘attribute–value’ pairs. We found that many attributes were entirely missing for a large proportion of samples, particularly key information on geographical locations. Consequently, several libraries were excluded due to the inability to assign BioSamples to Europe (in the case of Diptera, Coleoptera, Lepidoptera and Hymenoptera). The initial large survey on the selected libraries was conducted using ribosomal and COI coding sequences, which were chosen as targets due to their high conservation across phylogenetically related fungi and their presumed role as housekeeping genes. This strategy enabled the preliminary identification of a diverse group of fungal taxa, including potential symbionts, environmental contaminants and pathogens. However, due to the origin of the reference sequences, this approach should primarily favour the detection of fungi within the order Hypocreales, potentially overlooking other taxonomic groups. Although ribosomal RNA (rRNA) could have been considered an alternative molecular marker for this screening, it was ultimately excluded due to two main drawbacks. First, rRNA sequences tend to be highly conserved across fungal taxa, increasing the likelihood of matches to distantly related species. Secondly, in transcriptomic experiments, rRNA is often selectively depleted during library preparation via polyA enrichment, commercial rRNA depletion kits, or exosome‐based protocols, resulting in its under‐representation in RNA‐seq datasets.

Even after discarding the rRNA sequences as targets, considerable variability in the number of mapped reads was still evident among libraries from insects known to host YLSs. This could have led to false negatives, particularly when few libraries were available for a given species, or when all libraries were derived from the same tissue. For example, the 15 analysed SRA libraries derived from the cicada *Meimuna mongolica* were all obtained from RNA extracted from compound eyes. These organs probably contain a low number of YLS cells and/or the symbionts may not be transcriptionally active, resulting in mapping levels below the threshold, even though the insect is known to harbour a YLS (Zheng et al. 2017).

### 4.4 Discovery of Ophiocordyceps‐Related Symbionts Across Insect Lineages

The initial screening identified some non‐hemipteran species as potential YLS‐bearing insects, including those already known to host fungi in mutualistic relationships, such as *Drosophila* species (Chandler et al. 2012) the striped ambrosia beetle ( *Trypodendron lineatum* ) (Lehenberger et al. 2019) and the European spruce bark beetle (*Ips typographus*) (Cheng et al. 2023). In this study, however, we focused exclusively on yeast‐like endosymbionts phylogenetically related to fungi of the family Ophiocordycipitaceae. Accordingly, the second step of our pipeline aimed to exclude, as far as possible, insects associated with fungi in stable associations of distinct phylogenetic origins. Following the second step, most non‐hemipteran species that were positive after the initial analysis were eliminated, leaving only two species: *Leptochilus alpestris* (Hymenoptera) and *Lydus trimaculatus* (Coleoptera). To the best of our knowledge, no published studies have reported an association between these insects and *Ophiocordyceps*‐related YLSs. Further experimental evidence is required to substantiate these two associations, as well as the 41 potential new associations found in hemipteran species. Among the latter, the two heteropteran species, *Neogerris hesione* and *Hydrometra stagnorum*, represent outliers. These two species, in fact, are semi‐aquatic predators or scavengers that feed on small arthropods stranded on the water surface or trapped in surface films (Armisén and Khila 2022). In contrast, a common feature of hemipteran species obligately dependent on yeast‐like symbionts is a diet deficient in essential amino acids and nitrogen, as found in plant xylem and phloem. Based solely on bioinformatic data, we cannot completely exclude the possibility that some of these detections reflect incidental association or parasitism by fungi belonging to Ophiocordycipitaceae, rather than stable symbiotic relationships. However, given that in our analysis all negative controls were negative and only false negatives occurred, the insect species identified as positive are therefore likely to be genuinely associated with YLSs.

Both the literature and bioinformatic surveys indicate that insect–YLS associations appear to be relatively widespread in Hemiptera, with 160 species documented and 41 potential ones identified in this study. These associations are reported mainly within the families Cicadidae and Coccidae. Mining publicly available raw insect genomic data could reveal additional unexplored associations, although this would require substantially greater computational and storage resources.

In conclusion, despite its status as an obligate endosymbiont, StYLS appears to retain remarkable metabolic flexibility that may be critical for the survival of its insect host. Phylogenetic analyses, together with the presence of virulence‐related proteins, strongly support the origin of StYLS from free‐living *Ophiocordyceps*‐related fungi and suggest that it forms a monophyletic lineage with other YLSs. The potential monophyletic origin of YLSs facilitated the search for new candidate YLS‐insect associations through the newly developed bioinformatic pipeline. This pipeline offers a flexible and customizable framework for large‐scale mining of SRA datasets and can be readily adapted to investigate a wide range of biological questions, including the discovery of additional novel microbe associations across diverse host lineages.

The phylogenetic distribution of the association with YLSs across the order Hemiptera aligns with the hypothesis that YLS–insect symbioses originated through multiple independent domestication events rather than from a single ancestral lineage. By supplying essential nutritional and physiological functions, these symbionts likely facilitated the colonization of nutrient‐poor ecological niches and contributed to the evolutionary success of their insect hosts. Therefore, understanding the diversity, distribution and functional contributions of these microbial symbionts is fundamental for designing sustainable strategies for their management.

## Author Contributions

**Alessandro Cicerone:** investigation, writing – review and editing. **Simona Abbà:** conceptualization, investigation, funding acquisition, writing – original draft, data curation, supervision, software, formal analysis, methodology, writing – review and editing, project administration, validation, visualization, resources. **Marta Vallino:** conceptualization, funding acquisition, writing – review and editing, project administration, visualization, resources, investigation, validation, writing – original draft. **Luciana Galetto:** investigation, writing – review and editing, resources. **Simona Cirrincione:** investigation, resources, writing – review and editing. **Beatrice Aiuto:** investigation, resources, writing – review and editing. **Marika Rossi:** writing – original draft, writing – review and editing, conceptualization, investigation, resources, funding acquisition, visualization, methodology, validation, project administration, supervision, formal analysis.

## Funding

This work was supported by Ministero dell'Agricoltura, della Sovranità Alimentare e delle Foreste of Italy, Azione di ricerca n. 1 (D.M. 419782, 14/08/2023), for the Project “MICOTI,” CUP B17G23000320005.

## Conflicts of Interest

The authors declare no conflicts of interest.

## Supporting information


**Data S1:** Genome assemblies used for phylogenetic analysis. An asterisk (*) is used to distinguish the five YLS genomes from other fungal genomes.

**Data S2:** List of the StYLS transcripts used for the initial screening of insect transcriptomic SRA libraries through Magic‐BLAST. The table reports NCBI accession numbers and functional annotations for each transcript.

**Data S3:** KEGG orthology (KO) assignments predicted using KEGG KAAS. The predicted StYLS proteins were functionally annotated using the KEGG Automatic Annotation Server (KAAS). The table lists the KEGG parent pathway maps, corresponding KEGG orthology identifiers (K codes), and their functional descriptions with enzyme commission (EC) numbers. Numbers in parentheses after each pathway indicate the total number of KO entries associated with that pathway in KEGG.

**Data S4:** KEGG module assignments predicted using BlastKOALA. Functional annotation of the predicted StYLS proteins was performed using BlastKOALA, and KEGG Orthology (KO) identifiers were mapped to KEGG metabolic modules. The table lists KEGG module identifiers, corresponding K codes (KO identifiers), and functional descriptions with enzyme commission (EC) numbers. Numbers in parentheses indicate the total number of KO entries associated with the module in KEGG, and module completeness is reported as the number of steps detected in the genome relative to the total steps required for the module.

**Data S5:** Carbohydrate‐active enzyme (CAZyme) annotation summary based on dbCAN predictions. The predicted StYLS proteins with CAZyme domains are listed with their gene IDs and associated enzyme commission (EC) numbers (when available). ‘dbCAN_sub’, ‘HMMER’ and ‘DIAMOND’ columns report domain hits and sequence similarity to CAZyme families. SignalP prediction shows whether the protein is predicted to contain a signal peptide (Y = yes, N = no); numbers in brackets indicate the amino acid position of the signal paptide. The ‘Number of Tools’ column reports how many of the three annotation methods (dbCAN_sub, HMMER and DIAMOND) supported the prediction. Only the predictions supported by all three methods were considered for further analysis.

**Data S6:** Protein identification by DIA‐NN and annotation by BLASTP. The table summarizes protein groups detected in *Scaphoideus titanus*, including the number of sequences per group (‘N.Sequences’) and the number of proteotypic (‘unique’) sequences (‘N.Proteotypic.Sequences’). The column ‘R73S1BA_scapho101125.raw’ shows protein intensity values derived from DIA‐NN quantification. Protein annotations and their best BLASTP hits are listed in the ‘Description’ column, including protein name and source organism. The ‘Phylum’ column indicates the taxonomic classification of the best hit. Protein identifiers beginning with ‘Eva’ were derived from the *Euscelidius variegatus* genome, whereas those beginning with ‘sulcia’ correspond to proteins from the *Candidatus Karelsulcia* muelleri Eva_TO strain identified in *E. variegatus* . Identifiers beginning with ‘ophio’ represent proteins predicted from the newly sequenced StYLS. Proteins with GenBank accession numbers starting with ‘XP_’ originate from the *Macrosteles quadrilineatus* genome, whereas identifiers beginning with ‘AXI’ correspond to proteins from the *Cardinium* endosymbiont of *Sogatella furcifera* . All other codes were derived from the transcriptomic data of the *Scaphoideus titanus* holobiont and may therefore originate from the insect host as well as from its associated primary and secondary endosymbionts. Asterisks (*) indicate that no significant BLASTp hit was found (*E*‐value > 0.0001).

**Data S7:** Insect species reported to be associated with yeast‐like symbionts in the literature. This table lists insect species known to be associated with YLSs, along with their taxonomic classification including family, suborder and order. Relevant literature sources reporting these associations are provided in the ‘Citation’ column.

**Data S8:** Variability in the number of mapped reads across tissues in SRA accessions of *Nilaparvata lugens.* Each SRA library is listed with its accession number, the number of reads matching StYLS sequences in the first step, the country of origin, and the tissue source of the analysed samples.

**Data S9:** Variability in the number of mapped reads across life stages in *Ericerus pela*. Each SRA library is listed with its accession number, the number of reads matching StYLS sequences in the first step, the country of origin, the tissue source and life stages of the analysed samples.

**Data S10:** Identification of potential insect species hosting yeast‐like symbionts across five insect orders. NCBI transcriptomic Sequence Read Archive (SRA) libraries from insect species belonging to Coleoptera, Diptera, Lepidoptera, Hemiptera and Hymenoptera were analysed using a two‐step bioinformatic pipeline. The column ‘Number of matching reads (1st step)’ indicates the number of reads initially matching the 19 StYLS reference sequences, as reported by Magic‐BLAST, whereas the column ‘Number of Ophiocordycipitaceae‐related transcripts (2nd step)’ shows the number of transcripts assigned to this family after the second step (read assembly followed by BLASTx analysis). Information on tissue source is also provided. European countries are reported as the geographic origins of insect samples. Only for the order Hemiptera was the screening extended to include all publicly available transcriptomic SRA datasets with known geographic origin, as the literature indicates that this order comprises the hosts in which Ophiocordyceps‐allied yeast‐like symbionts are most likely to occur. For the Hemiptera subset, an additional column (‘Notes’) indicates whether the species has been reported in the literature to be associated with an Ophiocordyceps‐related YLS (positive control) or not associated with fungal endosymbionts (negative control). Species with read counts ≥ 20 after the first step are highlighted in bold; among these, those with transcript counts ≥ 23 in the second step are highlighted in green. If multiple libraries from the same species met the first‐step threshold, only the library with the highest number of matching reads was selected for the second step.

## Data Availability

The StYLS genome is available at NCBI under BioProject PRJNA1420485. The mass spectrometry proteomics data have been deposited to the ProteomeXchange Consortium via the PRIDE partner repository with the Project Accession: PXD075610 and Token: mcKfaqlxq2rp. The draft genome, the predicted proteome of StYLS, the assembled insect SRA libraries that passed the threshold of the first step of the bioinformatic workflow, and the corresponding BLASTx results are deposited in Zenodo: https://doi.org/10.5281/zenodo.18504810, https://doi.org/10.5281/zenodo.18506981, https://doi.org/10.5281/zenodo.18507008, https://doi.org/10.5281/zenodo.18506951.

## References

[emi70361-bib-0001] Abbà, S. , M. Rossi , M. Vallino , L. Galetto , C. Marzachì , and M. Turina . 2022. “Metatranscriptomic Assessment of the Microbial Community Associated With the Flavescence Dorée Phytoplasma Insect Vector Scaphoideus Titanus.” Frontiers in Microbiology 13: 866523. 35516423 10.3389/fmicb.2022.866523 PMC9063733

[emi70361-bib-0002] Abbà, S. , M. Vallino , S. Cirrincione , et al. 2025. “Rewiring the Proteome of the Euscelidius Variegatus Holobiont in Response to Flavescence Dorée Phytoplasma.” Scientific Reports 16: 1171. 41345534 10.1038/s41598-025-30920-7 PMC12789426

[emi70361-bib-0003] Ali, S. S. , J. Wu , R. Xie , F. Zhou , J. Sun , and M. Huang . 2017. “Screening and Characterizing of Xylanolytic and Xylose‐Fermenting Yeasts Isolated From the Wood‐Feeding Termite, Reticulitermes Chinensis.” PLoS One 12: e0181141. 28704553 10.1371/journal.pone.0181141 PMC5509302

[emi70361-bib-0004] Alonge, M. , L. Lebeigle , M. Kirsche , et al. 2022. “Automated Assembly Scaffolding Using RagTag Elevates a New Tomato System for High‐Throughput Genome Editing.” Genome Biology 23: 258. 36522651 10.1186/s13059-022-02823-7 PMC9753292

[emi70361-bib-0005] Armisén, D. , and A. Khila . 2022. “Genomics of the Semi‐Aquatic Bugs (Heteroptera; Gerromorpha): Recent Advances Toward Establishing a Model Lineage for the Study of Phenotypic Evolution.” Current Opinion in Insect Science 50: 100870. 34990871 10.1016/j.cois.2021.12.010

[emi70361-bib-0006] Asnicar, F. , A. M. Thomas , F. Beghini , et al. 2020. “Precise Phylogenetic Analysis of Microbial Isolates and Genomes From Metagenomes Using PhyloPhlAn 3.0.” Nature Communications 11: 2500. 10.1038/s41467-020-16366-7 PMC7237447 32427907

[emi70361-bib-0007] Baig, F. , K. Farnier , M. Ishtiaq , and J. P. Cunningham . 2023. “Volatiles Produced by Symbiotic Yeasts Improve Trap Catches of *Carpophilus davidsoni* (Coleoptera: Nitidulidae): An Important Pest of Stone Fruits in Australia.” Journal of Economic Entomology 116: 505–512. 36881679 10.1093/jee/toad027

[emi70361-bib-0008] Becher, P. G. , G. Flick , E. Rozpędowska , et al. 2012. “Yeast, Not Fruit Volatiles Mediate *Drosophila melanogaster* Attraction, Oviposition and Development.” Functional Ecology 26: 822–828.

[emi70361-bib-0009] Bellutti, N. , A. Gallmetzer , G. Innerebner , S. Schmidt , R. Zelger , and E. H. Koschier . 2018. “Dietary Yeast Affects Preference and Performance in Drosophila Suzukii.” Journal of Pest Science 91: 651–660. 29568250 10.1007/s10340-017-0932-2 PMC5847167

[emi70361-bib-0010] Bennett, G. M. , and N. A. Moran . 2013. “Small, Smaller, Smallest: The Origins and Evolution of Ancient Dual Symbioses in a Phloem‐Feeding Insect.” Genome Biology and Evolution 5: 1675–1688. 23918810 10.1093/gbe/evt118 PMC3787670

[emi70361-bib-0011] Blackwell, M. 2017. “Made for Each Other: Ascomycete Yeasts and Insects.” Microbiology Spectrum 5. 10.1128/microbiolspec.funk-0081-2016 PMC11687504 28597823

[emi70361-bib-0012] Boetzer, M. , C. V. Henkel , H. J. Jansen , D. Butler , and W. Pirovano . 2011. “Scaffolding Pre‐Assembled Contigs Using SSPACE.” Bioinformatics 27: 578–579. 21149342 10.1093/bioinformatics/btq683

[emi70361-bib-0013] Boratyn, G. M. , J. Thierry‐Mieg , D. Thierry‐Mieg , B. Busby , and T. L. Madden . 2019. “Magic‐BLAST, an Accurate RNA‐Seq Aligner for Long and Short Reads.” BMC Bioinformatics 20: 405. 31345161 10.1186/s12859-019-2996-x PMC6659269

[emi70361-bib-0014] Cantarel, B. L. , I. Korf , S. M. C. Robb , et al. 2008. “MAKER: An Easy‐To‐Use Annotation Pipeline Designed for Emerging Model Organism Genomes.” Genome Research 18: 188–196. 18025269 10.1101/gr.6743907 PMC2134774

[emi70361-bib-0015] Chandler, J. A. , J. A. Eisen , and A. Kopp . 2012. “Yeast Communities of Diverse Drosophila Species: Comparison of Two Symbiont Groups in the Same Hosts.” Applied and Environmental Microbiology 78: 7327–7336. 22885750 10.1128/AEM.01741-12 PMC3457106

[emi70361-bib-0016] Cheng, T. , T. Veselská , B. Křížková , et al. 2023. “Insight Into the Genomes of Dominant Yeast Symbionts of European Spruce Bark Beetle, *Ips Typographus* .” Frontiers in Microbiology 14: 1108975. 37077248 10.3389/fmicb.2023.1108975 PMC10106607

[emi70361-bib-0017] Chikhi, R. , T. Lemane , R. Loll‐Krippleber , et al. 2024. “Logan: Planetary‐Scale Genome Assembly Surveys Life's Diversity.” 10.1101/2024.07.30.605881.

[emi70361-bib-0018] Cong, J. , K. Xiao , W. Jiao , et al. 2022. “The Coupling Between Cell Wall Integrity Mediated by MAPK Kinases and SsFkh1 Is Involved in Sclerotia Formation and Pathogenicity of Sclerotinia Sclerotiorum.” Frontiers in Microbiology 13: 816091. 35547112 10.3389/fmicb.2022.816091 PMC9081980

[emi70361-bib-0019] Cooper, W. R. , W. B. Walker , G. M. Angelella , et al. 2023. “Bacterial Endosymbionts Identified From Leafhopper (Hemiptera: Cicadellidae) Vectors of Phytoplasmas.” Environmental Entomology 52: 243–253. 36869841 10.1093/ee/nvad015

[emi70361-bib-0020] Cornwallis, C. K. , A. van 't Padje , J. Ellers , et al. 2023. “Symbioses Shape Feeding Niches and Diversification Across Insects.” Nature Ecology & Evolution 7: 1022–1044. 37202501 10.1038/s41559-023-02058-0 PMC10333129

[emi70361-bib-0021] Ding, J.‐L. , K. Wei , M.‐G. Feng , and S.‐H. Ying . 2023. “Homologs of Bacterial Heat‐Labile Enterotoxin Subunit A Contribute to Development, Stress Response, and Virulence in Filamentous Entomopathogenic Fungus Beauveria Bassiana.” Frontiers in Immunology 14: 1264560. 37809075 10.3389/fimmu.2023.1264560 PMC10556748

[emi70361-bib-0022] Edgar, R. C. , B. Taylor , V. Lin , et al. 2022. “Petabase‐Scale Sequence Alignment Catalyses Viral Discovery.” Nature 602: 142–147. 35082445 10.1038/s41586-021-04332-2

[emi70361-bib-0023] Fan, H.‐W. , H. Noda , H.‐Q. Xie , Y. Suetsugu , Q.‐H. Zhu , and C.‐X. Zhang . 2015. “Genomic Analysis of an Ascomycete Fungus From the Rice Planthopper Reveals How It Adapts to an Endosymbiotic Lifestyle.” Genome Biology and Evolution 7: 2623–2634. 26338189 10.1093/gbe/evv169 PMC4607526

[emi70361-bib-0024] Gómez‐Gil, E. , A. Franco , B. Vázquez‐Marín , et al. 2021. “Specific Functional Features of the Cell Integrity MAP Kinase Pathway in the Dimorphic Fission Yeast Schizosaccharomyces Japonicus.” Journal of Fungi 7: 482. 34198697 10.3390/jof7060482 PMC8232204

[emi70361-bib-0025] Gomez‐Polo, P. , M. J. Ballinger , M. Lalzar , et al. 2017. “An Exceptional Family: *Ophiocordyceps* ‐Allied Fungus Dominates the Microbiome of Soft Scale Insects (Hemiptera: Sternorrhyncha: Coccidae).” Molecular Ecology 26: 5855–5868. 28833928 10.1111/mec.14332

[emi70361-bib-0026] Haas, B. 2023. “TransDecoder.”

[emi70361-bib-0027] Hill, K. B. R. , D. C. Marshall , K. Marathe , et al. 2021. “The Molecular Systematics and Diversification of a Taxonomically Unstable Group of Asian Cicada Tribes Related to Cicadini Latreille, 1802 (Hemiptera: Cicadidae).” Invertebrate Systematics 35: 570–601.

[emi70361-bib-0028] Hongoh, Y. , and H. Ishikawa . 2000. “Evolutionary Studies on Uricases of Fungal Endosymbionts of Aphids and Planthoppers.” Journal of Molecular Evolution 51: 265–277. 11029071 10.1007/s002390010088

[emi70361-bib-0029] Huang, Z. , D. Wang , J. Zhou , H. He , and C. Wei . 2024. “Segregation of Endosymbionts in Complex Symbiotic System of Cicadas Providing Novel Insights Into Microbial Symbioses and Evolutionary Dynamics of Symbiotic Organs in Sap‐Feeding Insects.” Frontiers in Zoology 21: 15. 38863001 10.1186/s12983-024-00536-0 PMC11165832

[emi70361-bib-0030] Karasikov, M. , H. Mustafa , D. Danciu , et al. 2025. “Efficient and Accurate Search in Petabase‐Scale Sequence Repositories.” Nature 647: 1036–1044. 41062695 10.1038/s41586-025-09603-w PMC12657231

[emi70361-bib-0031] Kobiałka, M. , A. Michalik , J. Szwedo , and T. Szklarzewicz . 2018. “Diversity of Symbiotic Microbiota in Deltocephalinae Leafhoppers (Insecta, Hemiptera, Cicadellidae).” Arthropod Structure & Development 47: 268–278. 29621609 10.1016/j.asd.2018.03.005

[emi70361-bib-0032] Kobiałka, M. , A. Michalik , M. Walczak , and T. Szklarzewicz . 2018. “Dual “Bacterial‐Fungal” Symbiosis in Deltocephalinae Leafhoppers (Insecta, Hemiptera, Cicadomorpha: Cicadellidae).” Microbial Ecology 75: 771–782. 28939987 10.1007/s00248-017-1075-y PMC5856902

[emi70361-bib-0033] Kobmoo, N. , D. Wichadakul , N. Arnamnart , R. C. Rodríguez De La Vega , J. J. Luangsa‐ard , and T. Giraud . 2018. “A Genome Scan of Diversifying Selection in *Ophiocordyceps* Zombie‐Ant Fungi Suggests a Role for Enterotoxins in Co‐Evolution and Host Specificity.” Molecular Ecology 27: 3582–3598. 30052297 10.1111/mec.14813

[emi70361-bib-0034] Koga, R. , and N. A. Moran . 2014. “Swapping Symbionts in Spittlebugs: Evolutionary Replacement of a Reduced Genome Symbiont.” ISME Journal 8: 1237–1246. 24401857 10.1038/ismej.2013.235 PMC4030230

[emi70361-bib-0035] Lehenberger, M. , P. H. W. Biedermann , and J. P. Benz . 2019. “Molecular Identification and Enzymatic Profiling of Trypodendron (Curculionidae: Xyloterini) Ambrosia Beetle‐Associated Fungi of the Genus Phialophoropsis (Microascales: Ceratocystidaceae).” Fungal Ecology 38: 89–97.

[emi70361-bib-0036] Ma, B. , H. Chang , M. Guo , et al. 2025. “Yeast‐Derived Volatiles Orchestrate an Insect‐Yeast Mutualism With Oriental Armyworm Moths.” Nature Communications 16: 1479. 10.1038/s41467-025-56354-3 PMC11811291 39929802

[emi70361-bib-0037] Malassigné, S. , G. Minard , L. Vallon , E. Martin , C. Valiente Moro , and P. Luis . 2021. “Diversity and Functions of Yeast Communities Associated With Insects.” Microorganisms 9: 1552. 34442634 10.3390/microorganisms9081552 PMC8399037

[emi70361-bib-0038] Manni, M. , M. R. Berkeley , M. Seppey , F. A. Simão , and E. M. Zdobnov . 2021. “BUSCO Update: Novel and Streamlined Workflows Along With Broader and Deeper Phylogenetic Coverage for Scoring of Eukaryotic, Prokaryotic, and Viral Genomes.” Molecular Biology and Evolution 38: 4647–4654. 34320186 10.1093/molbev/msab199 PMC8476166

[emi70361-bib-0039] Martinson, V. G. 2020. “Rediscovering a Forgotten System of Symbiosis: Historical Perspective and Future Potential.” Genes 11: 1063. 32916942 10.3390/genes11091063 PMC7563122

[emi70361-bib-0040] Matsuura, Y. , M. Moriyama , P. Łukasik , et al. 2018. “Recurrent Symbiont Recruitment From Fungal Parasites in Cicadas.” Proceedings of the National Academy of Sciences of the United States of America 115, no. 26: E5970–E5979. 29891654 10.1073/pnas.1803245115 PMC6042066

[emi70361-bib-0041] McCutcheon, J. P. , and N. A. Moran . 2010. “Functional Convergence in Reduced Genomes of Bacterial Symbionts Spanning 200 My of Evolution.” Genome Biology and Evolution 2: 708–718. 20829280 10.1093/gbe/evq055 PMC2953269

[emi70361-bib-0042] Meriggi, N. , M. Di Paola , F. Vitali , et al. 2019. “ *Saccharomyces* c*erevisiae* Induces Immune Enhancing and Shapes Gut Microbiota in Social Wasps.” Frontiers in Microbiology 10: 2320. 31681197 10.3389/fmicb.2019.02320 PMC6803456

[emi70361-bib-0043] Nishino, T. , M. Tanahashi , C.‐P. Lin , R. Koga , and T. Fukatsu . 2016. “Fungal and Bacterial Endosymbionts of Eared Leafhoppers of the Subfamily Ledrinae (Hemiptera: Cicadellidae).” Applied Entomology and Zoology 51: 465–477.

[emi70361-bib-0044] Noda, H. , and Y. Koizumi . 2003. “Sterol Biosynthesis by Symbiotes: Cytochrome P450 Sterol C‐22 Desaturase Genes From Yeastlike Symbiotes of Rice Planthoppers and Anobiid Beetles.” Insect Biochemistry and Molecular Biology 33: 649–658. 12770582 10.1016/s0965-1748(03)00056-0

[emi70361-bib-0045] Noda, H. , N. Nakashima , and M. Koizumi . 1995. “Phylogenetic Position of Yeast‐Like Symbiotes of Rice Planthoppers Based on Partial 18S rDNA Sequences.” Insect Biochemistry and Molecular Biology 25: 639–646. 7787846 10.1016/0965-1748(94)00107-s

[emi70361-bib-0046] Nurk, S. , D. Meleshko , A. Korobeynikov , and P. A. Pevzner . 2017. “metaSPAdes: A New Versatile Metagenomic Assembler.” Genome Research 27: 824–834. 28298430 10.1101/gr.213959.116 PMC5411777

[emi70361-bib-0047] Palmer, J. , and J. Stajich . 2020. “Funannotate v1.8.1: Eukaryotic Genome Annotation.”

[emi70361-bib-0048] Podsiadło, E. , K. Michalik , A. Michalik , and T. Szklarzewicz . 2018. “Yeast‐Like Microorganisms in the Scale Insect Kermes Quercus (Insecta, Hemiptera, Coccomorpha: Kermesidae). Newly Acquired Symbionts?” Arthropod Structure & Development 47: 56–63. 29126983 10.1016/j.asd.2017.11.002

[emi70361-bib-0049] Sacchi, L. , M. Genchi , E. Clementi , et al. 2008. “Multiple Symbiosis in the Leafhopper Scaphoideus Titanus (Hemiptera: Cicadellidae): Details of Transovarial Transmission of Cardinium sp. and Yeast‐Like Endosymbionts.” Tissue & Cell 40: 231–242. 18272191 10.1016/j.tice.2007.12.005

[emi70361-bib-0050] Sasaki, T. , M. Kawamura , and H. Ishikawa . 1996. “Nitrogen Recycling in the Brown Planthopper, *Nilaparvata lugens* : Involvement of Yeast‐Like Endosymbionts in Uric Acid Metabolism.” Journal of Insect Physiology 42: 125–129.

[emi70361-bib-0051] Sayers, E. W. , E. E. Bolton , A. M. Fine , et al. 2026. “Database Resources of the National Center for Biotechnology Information in 2026.” Nucleic Acids Research 54: D20–D27. 41385079 10.1093/nar/gkaf1060 PMC12807769

[emi70361-bib-0052] Stork, N. E. 2018. “How Many Species of Insects and Other Terrestrial Arthropods Are There on Earth?” Annual Review of Entomology 63: 31–45. 10.1146/annurev-ento-020117-043348 28938083

[emi70361-bib-0053] Suh, S.‐O. , H. Noda , and M. Blackwell . 2001. “Insect Symbiosis: Derivation of Yeast‐Like Endosymbionts Within an Entomopathogenic Filamentous Lineage.” Molecular Biology and Evolution 18: 995–1000. 11371588 10.1093/oxfordjournals.molbev.a003901

[emi70361-bib-0054] Szklarzewicz, T. , and A. Michalik . 2017. “Transovarial Transmission of Symbionts in Insects.” Results and Problems in Cell Differentiation 63: 43–67. 28779313 10.1007/978-3-319-60855-6_3

[emi70361-bib-0055] Szklarzewicz, T. , K. Michalik , B. Grzywacz , M. Kalandyk‐Kołodziejczyk , and A. Michalik . 2021. “Fungal Associates of Soft Scale Insects (Coccomorpha: Coccidae).” Cells 10: 1922. 34440691 10.3390/cells10081922 PMC8394295

[emi70361-bib-0056] Toki, W. 2021. “A Single Case Study of Mycetangia‐Associated Fungi and Their Abilities to Assimilate Wood‐Associated Carbon Sources in the Ship Timber Beetle *Elateroides flabellicornis* (Coleoptera: Lymexylidae) in Japan.” Symbiosis 83: 173–181.

[emi70361-bib-0057] Vaishally , S. Pal , K. R. Thyagarajan , and S. P. Shukla . 2025. “An Endosymbiotic Origin of the Crimson Pigment From the Lac Insect.” Proceedings of the National Academy of Sciences of the United States of America 122, no. 25: e2501623122. 40523179 10.1073/pnas.2501623122 PMC12207437

[emi70361-bib-0058] Valzania, L. , V. G. Martinson , R. E. Harrison , et al. 2018. “Both Living Bacteria and Eukaryotes in the Mosquito Gut Promote Growth of Larvae.” PLoS Neglected Tropical Diseases 12: e0006638. 29979680 10.1371/journal.pntd.0006638 PMC6057668

[emi70361-bib-0059] van Roosmalen, E. , and C. de Bekker . 2024. “Mechanisms Underlying Ophiocordyceps Infection and Behavioral Manipulation of Ants: Unique or Ubiquitous?” Annual Review of Microbiology 78: 575–593. 10.1146/annurev-micro-041522-092522 39270680

[emi70361-bib-0060] Vashishtha, A. , K. K. Sharama , and S. Lakhanpaul . 2011. “Co‐Existence, Phylogeny and Putative Role of Wolbachia and Yeast‐Like Symbiont (YLS) in Kerria Lacca (Kerr).” Current Microbiology 63: 206–212. 21674166 10.1007/s00284-011-9961-x

[emi70361-bib-0061] Vogel, K. J. , and N. A. Moran . 2013. “Functional and Evolutionary Analysis of the Genome of an Obligate Fungal Symbiont.” Genome Biology and Evolution 5: 891–904. 23563967 10.1093/gbe/evt054 PMC3673620

[emi70361-bib-0062] Wang, D. , Z. Huang , J. Billen , G. Zhang , H. He , and C. Wei . 2021. “Structural Diversity of Symbionts and Related Cellular Mechanisms Underlying Vertical Symbiont Transmission in Cicadas.” Environmental Microbiology 23: 6603–6621. 34390615 10.1111/1462-2920.15711

[emi70361-bib-0063] Wang, D. , Z. Huang , J. Billen , G. Zhang , H. He , and C. Wei . 2022. “Complex Co‐Evolutionary Relationships Between Cicadas and Their Symbionts.” Environmental Microbiology 24: 195–211. 34927333 10.1111/1462-2920.15829

[emi70361-bib-0064] Ward, C. M. , C. A. Onetto , and A. R. Borneman . 2024. “Adaptation During the Shift From Entomopathogen to Endosymbiont Is Accompanied by Gene Loss and Intensified Selection.” Genome Biology and Evolution 16, no. 12: evae251. 39561190 10.1093/gbe/evae251 PMC11632363

[emi70361-bib-0065] Weihrauch, D. , and M. J. O'Donnell . 2021. “Mechanisms of Nitrogen Excretion in Insects.” Current Opinion in Insect Science 47: 25–30. 33609767 10.1016/j.cois.2021.02.007

[emi70361-bib-0066] Xue, J. , X. Zhou , C.‐X. Zhang , et al. 2014. “Genomes of the Rice Pest Brown Planthopper and Its Endosymbionts Reveal Complex Complementary Contributions for Host Adaptation.” Genome Biology 15: 521. 25609551 10.1186/s13059-014-0521-0 PMC4269174

[emi70361-bib-0067] Yin, Y. , X. Mao , J. Yang , X. Chen , F. Mao , and Y. Xu . 2012. “dbCAN: A Web Resource for Automated Carbohydrate‐Active Enzyme Annotation.” Nucleic Acids Research 40: W445–W451. 22645317 10.1093/nar/gks479 PMC3394287

[emi70361-bib-0068] Zhao, X. , R. Mehrabi , and J.‐R. Xu . 2007. “Mitogen‐Activated Protein Kinase Pathways and Fungal Pathogenesis.” Eukaryotic Cell 6: 1701–1714. 17715363 10.1128/EC.00216-07 PMC2043402

[emi70361-bib-0069] Zheng, Z. , D. Wang , H. He , and C. Wei . 2017. “Bacterial Diversity of Bacteriomes and Organs of Reproductive, Digestive and Excretory Systems in Two Cicada Species (Hemiptera: Cicadidae).” PLoS One 12: e0175903. 28437427 10.1371/journal.pone.0175903 PMC5402938

[emi70361-bib-0070] Zhou, J. , Q. Guo , X. Han , et al. 2025. “Genome Degradation Results in Nested Symbiosis and Endosymbiont Replacement in Cicadas.” Nature Communications 16: 10104. 10.1038/s41467-025-65129-9 PMC12627099 41253823

