# Supplementary material for: Multi‐Omics Profiling of the Scaphoideus titanus Yeast‐Like Symbiont Guides the Bioinformatic Discovery of Related Fungal Symbioses in Insects
Source: Environ Microbiol. 2026 Jul 2;28(7):e70361. doi: 10.1111/1462-2920.70361 (PMC13327812; doi:10.1111/1462-2920.70361)
Supplement: Supplementary file 1 — Data S1: Genome assemblies used for phylogenetic analysis. An asterisk (*) is used to distinguish the five YLS genomes from other fungal genomes. [file EMI-28-e70361-s010.docx]

**Supplementary Material 1: Genome assemblies used for the phylogenetic analysis**. An asterisk (*) marks the five YLS genomes that are publicly available

| **NCBI Assembly Name** | **Assembly Accession** | **Organism Name** |
| --- | --- | --- |
| BasiARSEF4850_Illumina | GCA_053540845.1 | *Beauveria asiatica* |
| ASM28067v1 | GCF_000280675.1 | *Beauveria bassiana* ARSEF 2860 |
| BBO 1.0 | GCA_001636735.1 | *Beauveria brongniartii* RCEF 3172 |
| BgryMY11210_Illumina | GCA_053540745.1 | *Beauveria gryllotalpidicola* |
| BmimMY10362_Illumina | GCA_053540725.1 | *Beauveria mimosiformis* |
| BnamMY8738_Illumina | GCA_053539385.1 | *Beauveria namnaoensis* |
| BneoNHJ6243_Illumina | GCA_053539345.1 | *Beauveria neobassiana* |
| BthaMY3296_Illumina | GCA_053539325.1 | *Beauveria thailandica* |
| YLSCbra_1.0 | GCA_000372705.1 | *Cerataphis brasiliensis* yeast-like symbiont * |
| CLO192961 | GCA_902085965.1 | *Clonostachys rosea* |
| ISF 1.0 | GCA_001636725.1 | *Cordyceps fumosorosea* ARSEF 2679 |
| ASM5110311v1 | GCA_051103115.1 | *Cordyceps javanica* |
| CmilitarisCM01_v01 | GCA_000225605.1 | *Cordyceps militaris* CM01 |
| ASM162519v1 | GCA_001625195.1 | *Drechmeria coniospora* |
| ASM1308505v1 | GCA_013085055.1 | *Fusarium oxysporum* Fo47 |
| ASM95604v1 | GCA_000956045.1 | *Hirsutella minnesotensis* 3608 |
| ASM2036097v2 | GCA_020360975.2 | *Hirsutella rhossiliensis* |
| iisc_KLYLS | GCA_051170975.1 | *Hypocreales sp.* KLYLS * |
| ASM293905v1 | GCA_002939055.1 | *Hypocreales sp.* Mo6-1 * |
| Coph_MacrARSEF324_H1_v1.0 | GCF_019434415.1 | *Metarhizium acridum* |
| MAM 1.0 for version 1 of the Metarhizium album genome | GCA_000804445.1 | *Metarhizium album* ARSEF 1941 |
| ASM42698v1 | GCA_000426985.1 | *Metarhizium anisopliae* BRIP 53284 |
| ASM1342620v1 | GCA_013426205.1 | *Metarhizium brunneum* |
| MGU_1.0 | GCA_000814955.1 | *Metarhizium guizhouense* ARSEF 977 |
| ASM2010229v1 | GCA_020102295.1 | *Metarhizium humberi* |
| MAN_1.0 | GCA_000814975.1 | *Metarhizium hybridum* |
| NOR 1.0 | GCA_001636745.1 | *Metarhizium rileyi* |
| MAA 2.0 | GCF_000187425.2 | *Metarhizium robertsii* ARSEF 23 |
| ASM75842v1 | GCA_000758425.1 | *Nilaparvata lugens* yeast-like symbiont * |
| OphauB2 | GCA_002591415.1 | *Ophiocordyceps australis* |
| ASM333945v1 | GCA_003339455.1 | *Ophiocordyceps camponoti-leonardi (nom. inval.)* |
| Ophun1 | GCA_002591395.1 | *Ophiocordyceps camponoti-rufipedis* |
| ASM333941v1 | GCA_003339415.1 | *Ophiocordyceps camponoti-saundersi (nom. inval.)* |
| OPF BCC54312 improved | GCA_001633055.2 | *Ophiocordyceps polyrhachis-furcata* BCC 54312 |
| ASM44836v1 | GCA_000448365.1 | *Ophiocordyceps sinensis* CO18 |
| ASM127257v2 | GCA_001272575.2 | *Ophiocordyceps unilateralis* |
| ASM314460v1 | GCA_003144605.1 | *Purpureocillium lilacinum* |
| ASM2260516v1 | GCA_022605165.1 | *Purpureocillium takamizusanense* |
| ASM290118v1 | GCA_002901185.1 | *Tolypocladium capitatum* |
| Tophv1.0 | GCA_001189435.1 | *Tolypocladium ophioglossoides* CBS 100239 |
| ASM291650v1 | GCA_002916505.1 | *Tolypocladium paradoxum* |
|  | https://zenodo.org/records/10780696 | YLS *Parthenolecanium corni* * |
