# Supplementary material for: Multi‐Omics Profiling of the Scaphoideus titanus Yeast‐Like Symbiont Guides the Bioinformatic Discovery of Related Fungal Symbioses in Insects
Source: Environ Microbiol. 2026 Jul 2;28(7):e70361. doi: 10.1111/1462-2920.70361 (PMC13327812; doi:10.1111/1462-2920.70361)
Supplement: Supplementary file 2 — Data S2: List of the StYLS transcripts used for the initial screening of insect transcriptomic SRA libraries through Magic‐BLAST. The table reports NCBI accession numbers and functional annotations for each transcript. [file EMI-28-e70361-s002.docx]

**Supplementary Material 2. List of the StYLS transcripts used for the initial screening of insect transcriptomic SRA libraries through Magic-BLAST.** The table reports NCBI accession numbers and functional annotations for each transcript.

| **NCBI Accession** | **Description** |
| --- | --- |
| GJQP01014303.1 | 40S ribosomal protein S30 |
| GJQL01007044.1 | 60S ribosomal protein L38 |
| GJQL01014605.1 | 60S ribosomal protein L21 |
| GJQL01018087.1 | 60S ribosomal protein L27a |
| GJQP01004673.1 | 60S ribosomal protein L8B |
| GJQL01081484.1 | 40S ribosomal protein S4 |
| GJQL01085004.1 | 40S ribosomal protein S12 |
| GJQP01000063.1 | 40S ribosomal protein S1 |
| GJQP01000216.1 | 40S ribosomal protein S18 |
| GJQP01002155.1 | 40S ribosomal protein S15 |
| GJQP01013778.1 | 40S ribosomal protein S5 |
| GJQP01017633.1 | 60S ribosomal protein L32 |
| GJQP01071374.1 | 60S ribosomal protein L30 |
| GJQP01074608.1 | 60S ribosomal protein L31 |
| GJQQ01013182.1 | 60S ribosomal protein L5 |
| GJQQ01035485.1 | 60S ribosomal protein L33 |
| GJQQ01042299.1 | 40S ribosomal protein S14 |
| GJQQ01104611.1 | 40S ribosomal protein S24 |
| GJQL01050732.1 | cytochrome oxidase subunit I |
