# Supplementary material for: Multi‐Omics Profiling of the Scaphoideus titanus Yeast‐Like Symbiont Guides the Bioinformatic Discovery of Related Fungal Symbioses in Insects
Source: Environ Microbiol. 2026 Jul 2;28(7):e70361. doi: 10.1111/1462-2920.70361 (PMC13327812; doi:10.1111/1462-2920.70361)
Supplement: Supplementary file 8 — Data S8: Variability in the number of mapped reads across tissues in SRA accessions of Nilaparvata lugens. Each SRA library is listed with its accession number, the number of reads matching StYLS sequences in the first step, the country of origin, and the tissue source of the analysed samples. [file EMI-28-e70361-s007.docx]

**Supplementary Material 8: Variability in the number of mapped reads across tissues in SRA accessions of *Nilaparvata lugens.*** Each SRA library is listed with its accession number, the number of reads matching StYLS sequences in the first step, the country of origin, and the tissue source analyzed.

| **SRA accession** | **Number of matching reads (1st step)** | **Country** | **Tissue** |
| --- | --- | --- | --- |
| SRR7339787 | 616 | China | All body |
| SRR19225964 | 54 | China | antenna |
| SRR13493647 | 18 | China | brain |
| SRR19225948 | 79 | China | cuticular |
| SRR27842851 | 109 | China | egg |
| SRR19225974 | 1711 | China | fat body |
| SRR13005601 | 0 | China | Forewing buds |
| SRR10090677 | 7 | China | gut |
| SRR13958483 | 2 | China | head |
| SRR19257395 | 178 | China | Hindgut |
| SRR13005591 | 1 | China | Hindwing buds |
| SRR12474157 | 163 | China | insect |
| SRR10605392 | 129 | China | insect body |
| SRR13958482 | 69 | China | integument |
| SRR8189327 | 14 | China | Leg |
| SRR19257389 | 162 | China | Malpighian tubules |
| SRR19257392 | 44 | China | Midgut |
| SRR18002509 | 0 | China | nota |
| SRR19225945 | 34 | China | ovary |
| SRR19225955 | 290 | China | ovipositor |
| SRR19225958 | 1 | China | salivary gland |
| SRR5644065 | 27 | China | salivary glands |
| SRR19225968 | 14 | China | testis |
| SRR17179553 | 99 | China | The fourth instar nymph |
| SRR6361096 | 1 | China | thorax |
| SRR6370730 | 53 | China | total body |
| SRR21011512 | 12 | China | whole body |
| SRR19225975 | 575 | China | fat body |
| SRR19225976 | 1021 | China | fat body |
| SRR13958498 | 737 | China | fat body |
