# Supplementary material for: Multi‐Omics Profiling of the Scaphoideus titanus Yeast‐Like Symbiont Guides the Bioinformatic Discovery of Related Fungal Symbioses in Insects
Source: Environ Microbiol. 2026 Jul 2;28(7):e70361. doi: 10.1111/1462-2920.70361 (PMC13327812; doi:10.1111/1462-2920.70361)
Supplement: Supplementary file 9 — Data S9: Variability in the number of mapped reads across life stages in Ericerus pela. Each SRA library is listed with its accession number, the number of reads matching StYLS sequences in the first step, the country of origin, the tissue source and life stages of the analysed samples. [file EMI-28-e70361-s001.docx]

**Supplementary Material 9: Variability in the number of mapped reads across life stages in *Ericerus pela***. Each SRA library is listed with its accession number, the number of reads matching StYLS sequences in the first step, the country of origin, the tissue source and life stages of the analyzed samples.

| **SRA accession** | **Number of matching reads (1st step)** | **Country** | **Tissue** | **Life stages** |
| --- | --- | --- | --- | --- |
| SRR9617902 | 9 | China | body | early female adult |
| SRR9617905 | 777 | China | body | early female adult |
| SRR9617914 | 25 | China | body | early female adult |
| SRR9617904 | 1569 | China | body | first-instar larvae (female) |
| SRR9617908 | 1062 | China | body | first-instar larvae (female) |
| SRR9617915 | 20829 | China | body | first-instar larvae (female) |
| SRR9617906 | 1401 | China | body | first-instar larvae (male) |
| SRR9617907 | 2374 | China | body | first-instar larvae (male) |
| SRR9617912 | 1662 | China | body | first-instar larvae (male) |
| SRR9617903 | 17 | China | body | late female adult |
| SRR9617909 | 13 | China | body | late female adult |
| SRR9617901 | 34 | China | body | late female adult |
| SRR9617916 | 2462 | China | body | second-instar larvae (female) |
| SRR9617917 | 4320 | China | body | second-instar larvae (female) |
| SRR9617918 | 7627 | China | body | second-instar larvae (female) |
| SRR9617910 | 30387 | China | body | second-instar larvae (male) |
| SRR9617911 | 13258 | China | body | second-instar larvae (male) |
| SRR9617913 | 29731 | China | body | second-instar larvae (male) |
